# The Protective Impact of *Aronia melanocarpa* L. Berries Extract against Prooxidative Cadmium Action in the Brain—A Study in an In Vivo Model of Current Environmental Human Exposure to This Harmful Element

**DOI:** 10.3390/nu16040502

**Published:** 2024-02-09

**Authors:** Agnieszka Ruczaj, Małgorzata M. Brzóska, Joanna Rogalska

**Affiliations:** Department of Toxicology, Medical University of Bialystok, Adama Mickiewicza 2C Street, 15-222 Bialystok, Poland; agnieszka.ruczaj@sd.umb.edu.pl (A.R.); joanna.rogalska@umb.edu.pl (J.R.)

**Keywords:** *Aronia melanocarpa* L. berries extract, brain, cadmium, chokeberry extract, nervous tissue, neurotoxicity, oxidative–antioxidative balance, oxidative stress, polyphenolic compounds, protection

## Abstract

Cadmium (Cd) is a prooxidant that adversely affects human health, including the nervous system. As exposure of the general population to this heavy metal is inevitable, it is crucial to look for agents that can prevent the effects of its toxic action. An experimental model on female rats of current lifetime human exposure to cadmium (3–24-months’ treatment with 1 or 5 mg Cd/kg diet) was used to test whether low-level and moderate intoxication can exert a prooxidative impact in the brain and whether supplementation with a 0.1% extract from the berries of *Aronia melanocarpa* L. (Michx.) Elliott (AE; chokeberry extract) can protect against this action. Numerous parameters of the non-enzymatic and enzymatic antioxidative barrier, as well as total antioxidative and oxidative status (TAS and TOS, respectively), were determined and the index of oxidative stress (OSI) was calculated. Moreover, chosen prooxidants (myeloperoxidase, xanthine oxidase, and hydrogen peroxide) and biomarkers of oxidative modifications of lipids, proteins, and deoxyribonucleic acid were assayed. Cadmium dysregulated the balance between oxidants and antioxidants in the brain and led to oxidative stress and oxidative injury of the cellular macromolecules, whereas the co-administration of AE alleviated these effects. To summarize, long-term, even low-level, cadmium exposure can pose a risk of failure of the nervous system by the induction of oxidative stress in the brain, whereas supplementation with products based on aronia berries seems to be an effective protective strategy.

## 1. Introduction

Currently, diseases of the nervous system, including neurodegenerative disorders, are a serious problem, especially in developed countries [1,2]. The etiopathogenesis of these diseases is complex, and genetic, as well as environmental and lifestyle factors, including diet, are engaged in their development [3,4]. It is estimated that among the potential causes of Alzheimer’s disease, belonging to the most common neurodegenerative disorders, lifestyle and environmental conditions that involve xenobiotics such as heavy metals like cadmium (Cd), account for 30% [4,5]. Available data show that a proper diet and especially supplementation with natural products (e.g., algae, fish oil, or berries) rich in biologically active substances characterized by pro-health properties, which include but are not limited to antioxidants such as polyphenols, vitamins E and C, xanthophylls, β-carotene, and selenium, can be important factors in protecting against numerous disorders of health status, including neurodegenerative diseases [3,6,7,8,9].

Neurotoxic effects were noted in humans chronically exposed to cadmium [2,4,5,10,11,12], and this toxic heavy metal has been recognized to play a role in the etiopathogenesis of Alzheimer’s disease [2,4,5,12,13]. It was revealed that in patients suffering from this disease with a blood concentration of cadmium exceeding 0.6 μg/L, mortality was higher compared to those with a concentration equal to or lower than 0.3 μg/L [2,12]. Owing to the growing prevalence of neurodegenerative diseases [1] and the evidence that environmental exposure to cadmium may be a risk factor for their development [2,4,5,12], as well as unavoidable intoxication of the general population with this harmful element [5,10,11,14], it is essential to recognize the unfavourable effect of this xenobiotic on the nervous system at current levels of environmental exposure, as well as to find agents that can effectively protect against this impact.

The mechanism of cadmium neurotoxicity is unclear; however, oxidative stress (the state of imbalance between the processes of oxidation and reduction with the predomination of the former ones) seems to be its crucial pathway [5,15,16,17,18,19,20,21,22,23,24,25,26,27,28,29,30,31,32,33,34]. Destroying the oxidative–antioxidative balance in cells underlies the development of many pathological states, including nervous system diseases [10,35,36]. Although the kidney is the critical organ for cadmium during long-term exposure [37] and this xenobiotic’s penetration through the undamaged blood–brain barrier is low, the brain, due to its structure rich in lipids, low antioxidative potential, and high usage of oxygen, seems to be particularly vulnerable to oxidative damage induced by this prooxidant [5,10,38]. In numerous studies performed in experimental animals, it was revealed that treatment with cadmium led to the retention of this element in the brain and damage to the nervous tissue, including oxidative–antioxidative misbalance and oxidative changes [15,16,17,18,19,20,21,22,23,24,25,26,27,28,29,30,31,32,33,34], but these effects were noted at an exposure higher than current environmental human exposure. Thus, based on these findings, it is impossible to recognize if such effects would also occur at low intoxication comparable to current environmental exposure in developed countries. 

Although the growing number of epidemiological data studies suggests that the current exposure of the general population to cadmium is a risk factor for neurodegenerative diseases [2,4,5,12], both the pathways of its neurotoxicity and the risk of nervous system injury at low exposure levels are unknown. The mechanisms of this heavy metal neurotoxicity, including its influence on the oxidative–antioxidative status of the nervous tissue, cannot be evaluated in vital states in humans because of the impossibility of collection of the nervous tissue but these effects can be well studied in animal models. Moreover, when carrying out studies in experimental models we can eliminate the impact of other factors that may influence the values of measured parameters and this is especially important in the case of xenobiotics being common environmental pollutants, like cadmium. Knowledge of the possible mechanisms of cadmium neurotoxicity at low-level intoxication is also necessary to undertake studies aimed at evaluating effective ways of both protecting against this action and treatment of these effects.

As for the general population, the main source of exposure to cadmium is food [11]. It is reasonable to look for protective substances that can also be delivered with food products, especially natural compounds possessing antioxidative properties and capable of interacting with cadmium already in the gastrointestinal tract, decreasing its absorption. Thus, the attention of our research team has been focused on chokeberries (*Aronia melanocarpa* L. berries (Michx.) Elliott, Rosaceae) [39,40,41,42,43,44,45], which are rich in polyphenols known for their pro-health properties, including a strong antioxidative potential and the ability to form stable complexes with ions of toxic elements [7,43,46,47,48,49]. The research performed on, developed by our research group, an animal model of low- and moderate-level human exposure to cadmium during a lifespan (1 and 5 mg Cd/kg diet, respectively, for 3–24 months) revealed that supplementation with a 0.1% aqueous extract from the berries of *A. melanocarpa* (AE) prevented numerous effects of the unfavourable impact of this toxic heavy metal, or at least attenuated their development [39,40,41,42,43,44,45]. Supplementation with this extract protected against the damaging impact of cadmium, including its action via oxidative stress, on the liver, kidney, bone tissue, and salivary glands [39,40,41,42,43,44,45,50]. The administration of AE also diminished cadmium accumulation in various organs, including the brain (Table S1) [39,40], as well as counteracted disturbances in the body status of some bioelements, including manganese [40].

Taking into account the finding that treatment with cadmium at concentrations of 1 and 5 mg Cd/kg diet resulted in oxidative stress in different organs [39,40,41,42,43,44,45,50], and in particular, the sensitivity of the brain to destroying its oxidative–reductive balance [5,10,38], it was justified to hypothesize that low-to-moderate long-term exposure to this toxic element will lead to the development of oxidative stress in the nervous tissue. Moreover, owing to the antioxidative potential of aronia berries [46,47] and the above-mentioned results regarding the amelioration of cadmium toxicity by supplementation with AE [39,40,41,42,43,44,45,50], including especially the lower retention of this xenobiotic in the brain (Table S1) [39,40] and the protection from the xenobiotic-caused changes of the status of manganese and the bioelement-dependent mitochondrial superoxide dismutase (MnSOD) in this organ [38], it has been hypothesized that the administration of AE can protect against oxidative stress and oxidative modifications of macromolecules caused by cadmium in the nervous tissue and in this way counteract its neurotoxicity. To verify the above-described hypotheses, numerous parameters of the oxidative–antioxidative status, the degree of oxidative stress, and damage to proteins, lipids, and deoxyribonucleic acid (DNA) in nervous tissue of brain origin were estimated. Similar studies have not been performed previously.

## 2. Materials and Methods

### 2.1. Cadmium in the Diet

To obtain diets that contained cadmium at concentrations of 1 and 5 mg/kg, cadmium chloride 2.5-hydrate (CdCl_2_ × 2.5 H_2_O; POCh, Gliwice, Poland) was added to the standard Labofeed H and Labofeed B diets (breeding diet and maintenance diet, respectively) at the stage of their production by Label Food “Morawski” (Kcynia, Poland). The compatibility of the cadmium concentration in the diets with the certified values (1 and 5 mg Cd/kg) was confirmed by analyses performed in our laboratory. The quantified values were 1.09 ± 0.13 and 4.92 ± 0.53 mg Cd/kg (mean ± standard deviation—SD) in the 1 and 5 mg Cd/kg diet, respectively [39].

### 2.2. The Extract from A. melanocarpa Berries

The *A. melanocarpa* fruit extract used in this study was delivered by Adamed Consumer Healthcare (Tuszyn, Poland) in the form of a lyophilized homogeneous powder, dark cherry to purple–red in colour, with a tart fruit taste and a faint, wine–fruity odour (typical of chokeberry fruits). The extract was microbiologically pure and water-soluble. According to the declaration of the producer (Certificate KJ 4/2010; Butch No. M100703), the content of polyphenolic compounds and anthocyanins in the extract reached 65.74% and 18.65%, respectively. The performed by our research group quantitative analysis of the ingredients of the commercial chokeberry extract revealed that the total concentration of polyphenols was 612.40 ± 3.33 mg/g, and the main groups of polyphenolic compounds included anthocyanins (202.28 ± 1.28 mg/g), proanthocyanidins (129.87 ± 1.12 mg/g), phenolic acids (110.92 ± 0.89 mg/g), and flavonoids (21.94 ± 0.98 mg/g) [41]. The polyphenolic profile of the powdered extract (estimated with the use of ultra performance liquid chromatography) has already been reported [41] and is available at https://www.mdpi.com/article/10.3390/ijms241411647/s1 (accessed on 10 January 2024) [45]. According to the producer, the extract also contained sugar, sugar alcohols (parasorboside and sorbitol), pectins, triterpenes, phytosterols, carotenoids, vitamins, and minerals. The chemical composition of chokeberries is widely described [47,48,49].

### 2.3. Experimental Animals

In this experiment, 192 young (at the age of 3–4 weeks) female Wistar rats (Hannover Wistar rats, that were bred according to the Charles River International Genetic Standardization Program—Crl: WI (Han)) were used. The animals were provided by certified breeding in Brwinów (Poland). The acclimatization of the rats to the laboratory conditions before the research lasted for 5 days. During the acclimatization, the animals were fed with the standard Labofeed H diet (Label Food “Morawski”, Kcynia, Poland) and redistilled water ad libitum. At the end of the acclimatization, there were no abnormalities in the animals’ health, and thus all females were included in the experiment. Throughout the experiment, the females were kept in a controlled, conventional environment, with artificial lighting (12 h light and 12 h dark) at a temperature of 22 ± 2 °C and a relative humidity of 50 ± 10%. The animals had unrestricted access to the Labofeed H diet during the first 3 months and the Labofeed B diet thereafter with and without cadmium addition and drinking fluids (redistilled water with or without the addition of powdered *A. melanocarpa* berries extract), as described below. Approval No. 60/2009 of the Local Ethics Committee for Animal Experiments in Bialystok (Poland) was obtained (21 September 2009).

### 2.4. Design of the Study

**Figure 1:**
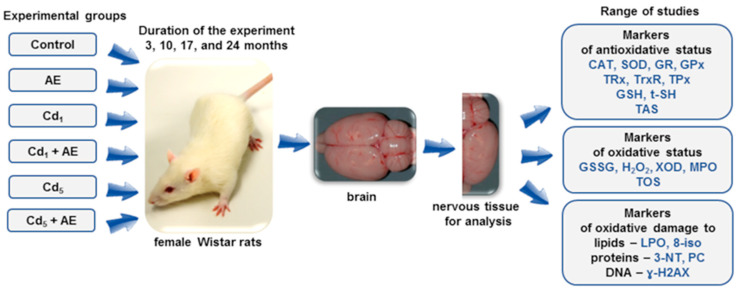
Schematic presentation of the experimental model and range of measurements performed in the present study ^1,2^. Female Wistar rats were administered commercial Labofeed diets that contained 1 or 5 mg cadmium (Cd)/kg (the Cd_1_ and Cd_5_ group, respectively) and/or 0.1% aqueous *A. melanocarpa* L. berries extract (AE, Cd_1_ + AE, and Cd_5_ + AE groups, respectively) from 3 up to 24 months. The rats from the control group received the standard Labofeed diet. CAT, catalase; DNA, deoxyribonucleic acid; GPx, glutathione peroxidase; GR, glutathione reductase; GSH, reduced glutathione; GSSG, oxidized glutathione; H_2_O_2_, hydrogen peroxide; MPO, myeloperoxidase; LPO, lipid peroxides; PC, protein carbonyl groups; SOD, superoxide dismutase; TAS, total antioxidative status; TOS, total oxidative status; TPx, thioredoxin peroxidase; t-SH, total thiol groups; Trx, thioredoxin; TrxR, thioredoxin reductase; XOD, xanthine oxidase; ɣ-H2AX, ɣ-H2A histone family member X; 3-NT, 3-nitrotyrosine; 8-iso, 8-isoprostane. ^1^ Apart from the measured parameters, the GSH/GSSG ratio and oxidative stress index (OSI = TOS/TAS) were calculated. ^2^ All photos presented in this figure come from the authors’ collection.

The rats were randomly allocated into 6 groups, with 32 animals in every group (mean body weight of about 65 g) (Figure 1). Two groups were given cadmium alone in the Labofeed diet at the concentrations of 1 mg/kg (the Cd_1_ group) or 5 mg/kg (the Cd_5_ group). One group received 0.1% aqueous AE alone, as the only drinking fluid (the AE group), and two groups were administered 0.1% AE during the feeding with the Labofeed diets that contained 1 or 5 mg Cd/kg (the Cd_1_ + AE group and Cd_5_ + AE group, respectively). The last group, which received the standard Labofeed diet containing 0.0584 ± 0.0049 mg Cd/kg and redistilled water (the content of cadmium was less than 0.05 μg Cd/L), served as a control group. More details on this experimental female rat model have been provided in previous publications [39,40,41,42,43,44,45].

The administration of the diet containing 1 mg Cd/kg corresponded to the current low-level general population exposure to this toxic heavy metal during a lifetime in well-developed countries, whereas the nourishment with the 5 mg Cd/kg diet reflected moderate environmental exposure [39]. The mean daily intake of cadmium throughout the whole duration of the experiment ranged from 39.2 to 83.8 µg/kg body weight (b.w.) and from 37.5 to 84.9 µg/kg b.w. in the animals from the Cd_1_ and Cd_1_ + AE groups, respectively, and reached 210.1–403.2 and 200.2–401.9 µg/kg b.w. in those from the Cd_5_ group and Cd_5_ + AE group, respectively [39]. The cadmium concentration in the blood of the rats was 0.113–0.324 and 0.735–1.332 μg/L in the Cd_1_ and Cd_5_ groups, respectively, whereas in the urine it was 0.085–0.285 and 0.284–0.695 μg/g creatinine, respectively [39] and it corresponded to the concentrations of this element noted in the general population of the countries around the world [5,14,51]. The blood cadmium concentration in the animals co-administered AE reached 0.117–0.305 and 0.584–1.149 μg/L in the Cd_1_ + AE and Cd_5_ + AE groups, respectively, whereas its concentration in the urine was 0.135–0.385 and 0.341–0.820 μg/g creatinine, respectively [39].

The 0.1% AE with which the animals were supplemented was prepared every day of the 24-month study by dissolution of 1 g of the commercial powdered chokeberry extract in 1 L of water (redistilled). The concentration of total polyphenolic compounds in the 0.1% AE was 612.4 ± 3.33 μg/mL (total anthocyanins—202.28 ± 1.28 μg/mL, including cyanidin 3-*O*-α-galactoside—80.07 ± 1.05 μg/mL, cyanidin 3-*O*-α-arabinoside—33.21 ± 0.01 μg/mL, and cyanidin 3-*O*-β-glucoside—3.68 ± 0.01 μg/mL, total proanthocyanidins—129.87 ± 1.12 μg/mL, total phenolic acids—110.92 ± 0.89 μg/mL, total flavonoids—21.94 ± 0.98 μg/mL and chlorogenic acid—68.32 ± 0.08 μg/mL) [41], while its cadmium concentration was <0.05 ng/mL [39].

The intake of the AE reached 67.4–146.6, 67.2–154.7, and 63.1–150.3 mg/kg b.w./24 h in the AE, Cd_1_ + AE, and Cd_5_ + AE groups, respectively, and the intake of polyphenolic compounds was within the following ranges: 44.3–96.4, 44.2–101.7, and 41.5–98.8 mg/kg b.w./24 h, respectively [39]. The consumption of the extract and polyphenolic compounds in the three experimental groups was independent of whether the extract was administered alone or under exposure to cadmium [39]. The 24 h intake of polyphenols by the female rats was from 2.9 to 7.3 times higher compared to their average supply noted in humans (1000 mg/24 h [52]). This research was conducted on females as they are more susceptible than males to the toxicity of cadmium due to its higher absorption from the digestive system and lower levels of iron in the body [53]. The 24-month duration of the experiment covered post-weaning maturation, growth into adulthood, and the stage of adulthood up to the elderly stage of the animals and it reflects about 60–70 years of human life [54,55]. Performing the planned measurements after 3, 10, 17, and 24 months made it possible to assess the effects of cadmium and/or AE in different periods of life, i.e., in youth, adulthood, and the elderly.

Throughout the whole experiment, no evidence of morbidity (including signs of a destroying impact on the nervous system) was observed; however, between the 17th and 24th month, three cases of spontaneous death were noted (one female from the group administered AE alone, as well as one each from the Cd_1_ and Cd_5_ groups) [39]. At the commencement of the particular periods of the study, i.e., after 3, 10, 17, and 24 months, 8 animals (this number of animals was sufficient to conduct a statistical analysis of the results) from each experimental group (with the exception of 7 rats in the AE, Cd_1_, and Cd_5_ groups after the 24-month study duration) were subjected to anaesthesia by the intraperitoneal administration of Morbital (Biowet, Pulawy, Poland) at a dose of 30 mg/kg b.w. and various organs and tissues, including the brain, were collected. The brain was evaluated macroscopically, gently dried with the use of filter paper, and weighed. If the biological material was not analysed without delay after collection, it was stored frozen at a temperature of −70 °C before the performance of all planned analyses.

### 2.5. Analytical Procedures

#### 2.5.1. Preparation of the Brain Homogenates

The brain of all animals from each group was sectioned into two parts (in the longitudinal plane as presented in Figure 1), which comprised all structures of this organ. Halves of the brain of known weight, about 0.7–1.1 g (an accuracy of weighing 0.0001 g), were homogenized in potassium phosphate buffer (pH = 7.4) with the addition of 10 μL of 0.5 M butylhydroxytoluene (Merck, Darmstadt, Germany) in acetonitrile (Merck, Darmstadt, Germany) per 1 mL of the homogenate (to prevent autooxidation) and use of the homogenizer Schütt Homogenplus (Labortechnik GmbH, Göttingen, Germany). The homogenates (20% weight/ volume) were then centrifuged (3000× *g*, 4 °C, 10 min) and aliquots were collected and kept at a temperature of −70 °C until the determination of all parameters.

#### 2.5.2. Establishment of the Markers of Oxidative–Antioxidative Balance and the Extent of Oxidative Stress and Oxidative Damage to Macromolecules in the Brain

To determine the balance between the processes of oxidation and reduction in the brain tissue, the activities of the key antioxidative enzymes, i.e., superoxide dismutase (SOD), catalase (CAT), glutathione reductase (GR), and glutathione peroxidase (GPx), and indices of the thioredoxin (Trx)-dependent antioxidative system, such as the concentrations of Trx, thioredoxin reductase (TrxR), and thioredoxin peroxidase (TPx), were determined. The concentrations of reduced glutathione (GSH), being the main non-enzymatic antioxidant in the body, and total thiol groups (t-SH) were assayed as well. The oxidized glutathione (GSSG) concentration was quantified to estimate the GSH/GSSG ratio in the brain. Total oxidative status and total antioxidative status (TOS and TAS, respectively) were determined and their ratio (TOS/TAS) was calculated as the index of oxidative stress (OSI). Prooxidants such as hydrogen peroxide (H_2_O_2_), myeloperoxidase (MPO), and xanthine oxidase (XOD) were measured as well. Moreover, markers of oxidative injury of lipids (concentrations of 8-isoprostane—8-iso and lipid peroxides–LPO), protein (concentrations of 3-nitrotyrosine–3-NT and protein carbonyl groups–PC), and DNA (concentrations of ɣ-H2A histone family member X—ɣ-H2AX) were established.

All of the analyses were performed using commercial spectrophotometric or enzyme-linked immunosorbent assay (ELISA) kits, except for CAT [56] and t-SH [57]. To determine the assayed variables, the spectrophotometers Epoch (Bio Tek Instruments, Inc., Winooski, VT, USA), MULTISCAN GO (Thermo Scientific, Vantaa, Finland), and Specord 50 Plus (Analityk Jena, Jena, Germany) were used. The intra- and inter-assay coefficients of variation (CV) were calculated to express the precision of particular measurements (the intra-assay CV reflects precision within an assay, e.g., an assay with the use of one kit, whereas the inter-assay CV is the precision between assays e.g., between two kits).

The values of all parameters determined in the brain were adjusted for total protein concentration assayed (CV < 3%) using a BioMaxima kit (No. 1-055-0200) purchased from Lublin (Poland).

##### Antioxidative Enzymes

SOD activity was assayed with the use of the Superoxide Dismutase Assay Kit (No. 706002) by the Cayman Chemical Company (Ann Arbor, MI, USA). To detect superoxide radicals that were generated by xanthine oxidase and hypoxanthine, tetrazolium salt was used in the method. The activity of CAT was quantified according to the Aebi method [56], based on H_2_O_2_ degradation by CAT, and the amount of undecomposed H_2_O_2_ was measured spectrophotometrically. The intra-assay CV for the first and the second used SOD kits was <2.5% and <5% and the inter-assay CV was <4%. The CV for CAT was <3%. GPx and GR activities were measured using commercial kits purchased from OxisResearch (Burlingame, CA, USA)—the Bioxytech GPx-340 (No. 21017) and Bioxytech GR-340 (No. 21018), respectively. To quantify the activity of GPx, a homogenate of the brain was added to a solution of GR, GSH, and reduced nicotinamide adenine dinucleotide phosphate (NADPH). After the addition of substrate (tert-butyl hydroperoxide), the absorbance was measured and it was proportional (directly) to the enzyme activity. The measurement of the activity of GR was based on the determination of the consumption of NADPH in the reaction of its oxidation to nicotinamide adenine dinucleotide phosphate (NADP^+^), which was catalysed by the limiting concentration of GR. The GPx and GR intra-assay CV were <5% and <3%, respectively.

##### Indices of Trx-Dependent Antioxidative System

Trx and TrxR concentrations were determined using the Rat (Trx) ELISA Kit (No. 201-11-0445) and Rat (TrxR) ELISA Kit (No. 201-11-0446) by SunRed (Shanghai, China), whereas the TPx concentration was assayed using the Rat Prdx2 (Peroxiredoxin-2) ELISA Kit (No. ER0450) by FineTest (Wuhan, China), which was based on the double-antibody sandwich ELISA technique. The intra-assay CV for Trx for the first kit reached <3% and <5% for the second one, whereas the inter-assay CV was <8%. The intra-assay CV for TrxR was <2% for the first kit and <3% for the second kit, and the inter-assay CV was <6%. In the case of TPx, the intra-assay CV was <2.5% and <1% for the two used kits, whereas the inter-assay CV was <9%.

##### GSH, GSSG, and t-SH

GSH and GSSG concentrations were established with the Glutathione Assay Kit (No. 703002) by Cayman Chemical Company (Ann Arbor, MI, USA). In this assay, the enzymatic recycling method with the use of GR was performed to determine the concentration of GSH. To quantify the concentration of GSSG, GSH was firstly derivatized with 2-vinylpyridine. The GSH intra-assay CV was <6% and <5% for the first kit and the second kit and the inter-assay CV was <2%. The intra-assay CV for the GSSG measurement was <6% for the first kit and <3% for the second one, and the inter-assay CV was <3%.

t-SH concentration was quantified according to Ellman’s method [57] based on the thiol groups (-SH groups)-dependent reduction of 5,5′-dithiobis(2-nitrobenzoic acid) to 2-nitro-5-chlorobenzoic acid. The intensity of absorbance of the product of this reaction was measured spectrophotometrically at 412 nm. The CV for the measurement of t-SH was <1%.

##### TOS and TAS

TOS and TAS were measured using spectrophotometric kits purchased from Immundiagnostik AG (Bensheim, Germany)—the PerOx (TOS/TOC) Kit (No. KC5100) and ImAnOx (TAS/TAC) Kit (No. KC5200), respectively. To determine TOS, total lipid peroxides present in the aliquot of the homogenate of the brain tissue were quantified in the reaction of peroxidase and peroxides present in the sample and the transformation of 3,3′,5,5′-tetramethylbenzidine added to the sample into a coloured product. The value of TAS was evaluated based on the reaction of antioxidant agents inherent in the homogenate aliquot and the added H_2_O_2_. The reference TOS values in the control samples included in the PerOx (TOS/TOC) Kit were 170–283 and 437–728 μmol/L for the first kit and 185–308 and 385–641 μmol/L for the second one. The TOS values assayed by us were 205.4 ± 9.7 and 618 ± 31.4 μmol/L, respectively, for the first kit and 233 ± 14.0 and 490 ± 25.2 μmol/L for the second kit. The inter-assay CV for TOS was <8%. TAS values in the samples included in the ImAnOx (TAS/TAC) Kit were 162–220 and 204–276 μmol/L. The values measured by us (188 ± 11.9 and 227 ± 10.7 μmol/L for the first kit, respectively, and 195 ± 13.7 μmol/L and 232 ± 11.4 μmol/L for the second kit, respectively) agreed with the certified values. The inter-assay CV for TAS was <2.6%.

##### H_2_O_2_, MPO, and XOD

The concentration of H_2_O_2_ was established colorimetrically using, provided by Oxis International, Inc. (Portland, OR, USA), the Bioxytech H_2_O_2_-560 kit (No. 21024). The method was based on the H_2_O_2_-induced iron(II) to iron(III) oxidation. The CV for H_2_O_2_ was <8%. MPO and XOD concentrations were measured with the use of double-antibody sandwich ELISA kits purchased from SunRed (Shanghai, China)—the Rat (MPO) ELISA kit (No. SRB-T-88360) and Rat (XOD) ELISA kit (No. 201-11-0575), respectively. The intra-assay CV for MPO measurements was <6% and <8% for the first and second kits, respectively, and the inter-assay CV was <10%. The intra-assay CV for XOD was <9% and <4% for the first and the second kit, respectively. The inter-assay CV for XOD was <4%.

##### Biomarkers of Oxidative Modifications of Lipids, Proteins, and DNA

The LPO concentration was established using the Bioxytech LPO-586 kit (No. 21012) purchased from OxisResearch (Burlingame, CA, USA) based on the reaction of N-methyl-2-phenylindole (a chromogenic reagent), malondialdehyde (MDA), and 4-hydroxyalkenals and the establishment of the absorbance of the formed stable chromophore. To establish the concentration of 8-iso, the Cayman Chemical Company (Ann Arbor, MI, USA) 8-Isoprostane ELISA Kit (No. 516351), in which 8-iso competed with 8-isoprostane-acetylcholinesterase conjugate for specific antiserum with a limited number of binding sites, was used. The absorption of the final product of this reaction was quantified spectrophotometrically and its intensity was proportional to the concentration of 8-iso. The CV for LPO was <6%. The intra-assay CV for the measurement of 8-iso was <4% and <3% for the first and the second kit, respectively, whereas the inter-assay CV reached <7%.

To assay the 3-NT concentration, the Nitrotyrosine (NT) ELISA Kit (No. CEB863Ge) by Cloud-Clone Corp. (Katy, TX, USA) was applied. In this test, the competitive inhibition ELISA technique was used. The PC concentration was measured using the Protein Carbonyl Assay Kit (No. 10005020) bought from the Cayman Chemical Company (Ann Arbor, MI, USA). The principle of this method is the reaction of 2,4-dinitrophenylhydrazine with PC and the spectrophotometric evaluation of the absorbance of the formed product. To establish the concentration of ɣ-H2AX, the Rat ɣ H2A Histone Family Member X (GH2AX) ELISA Kit (No. E02G0567) by BlueGene Biotech (Shanghai, China) was used. It is based on the competition of GH2AX from the assayed sample and the GH2AX-HRP conjugate for the anti-GH2AX antibody binding site. The intensity of the colour of the final product (inversely proportional to the concentration of ɣ-H2AX) was measured spectrophotometrically. The intra-assay CV for 3-NT was <6% and <4% for the first and second kits, respectively. The inter-assay CV for 3-NT was <10%. The intra-assay CV for PC was <3%. The intra-assay CV for ɣ-H2AX was <3% for the first kit and <5% for the second kit, while the inter-assay CV was <5%.

#### 2.5.3. Statistical Analysis

**Figure 2:**
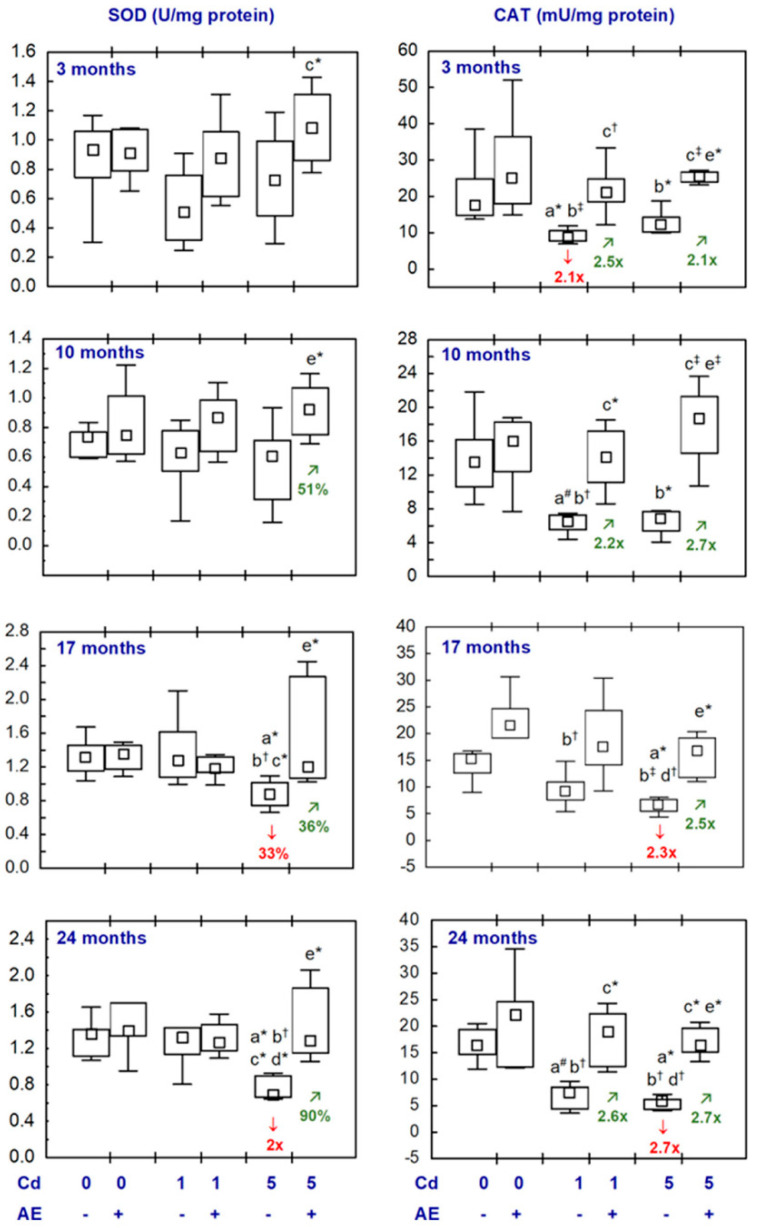
The activities of superoxide dismutase (SOD) and catalase (CAT) in the brains of female rats treated with cadmium (Cd) (0, 1, or 5 mg Cd/kg diet) and/or 0.1% extract from *A. melanocarpa* L. berries (AE). Statistical differences from the: a—Control group, b—AE group, c—Cd_1_ group, d—Cd_1_ + AE group, and e—Cd_5_ group are marked as * *p* < 0.05, ^†^ *p* < 0.01, ^‡^ *p* < 0.001, and ^#^ *p* = 0.07–0.08. The differences in comparison to the control animals (↓, lower) and the appropriate Cd group (↗, higher) are marked with the numerical values indicating a percentage difference or a fold of difference between the respective groups. The effect size (η^2^) for the differences in the activities of SOD and CAT between the experimental groups was large (0.176–0.330 and 0.595–0.624, respectively). Detailed data on the activities of both enzymes (including η^2^) are provided in Table S3.

**Figure 3:**
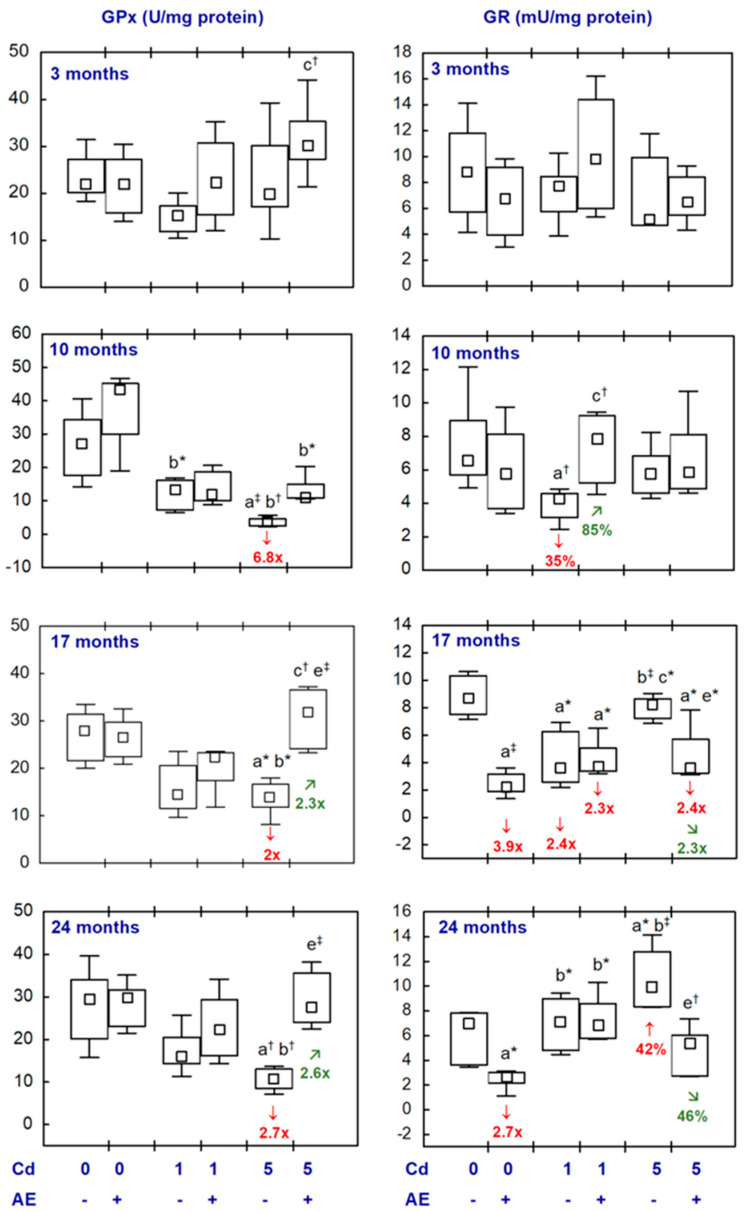
The activities of glutathione peroxidase (GPx) and glutathione reductase (GR) in the brains of female rats treated with cadmium (Cd) (0, 1, or 5 mg Cd/kg diet) and/or 0.1% extract from *A. melanocarpa* L. berries (AE). Statistical differences from the: a—Control group, b—AE group, c—Cd_1_ group, and e—Cd_5_ group are marked as * *p* < 0.05, ^†^ *p* < 0.01, and ^‡^ *p* < 0.001. The differences in comparison to the control animals (↑, higher and ↓, lower) and the appropriate Cd group (↗, higher and ↘, lower) are marked with the numerical values indicating a percentage difference or a fold of difference between the respective groups. The effect size (η^2^) for the differences in the activities of GPx and GR between the experimental groups was large (0.278–0.730 and 0.270–0.709, respectively). Detailed data on the activities of both enzymes (including η^2^) are provided in Table S4.

**Figure 4:**
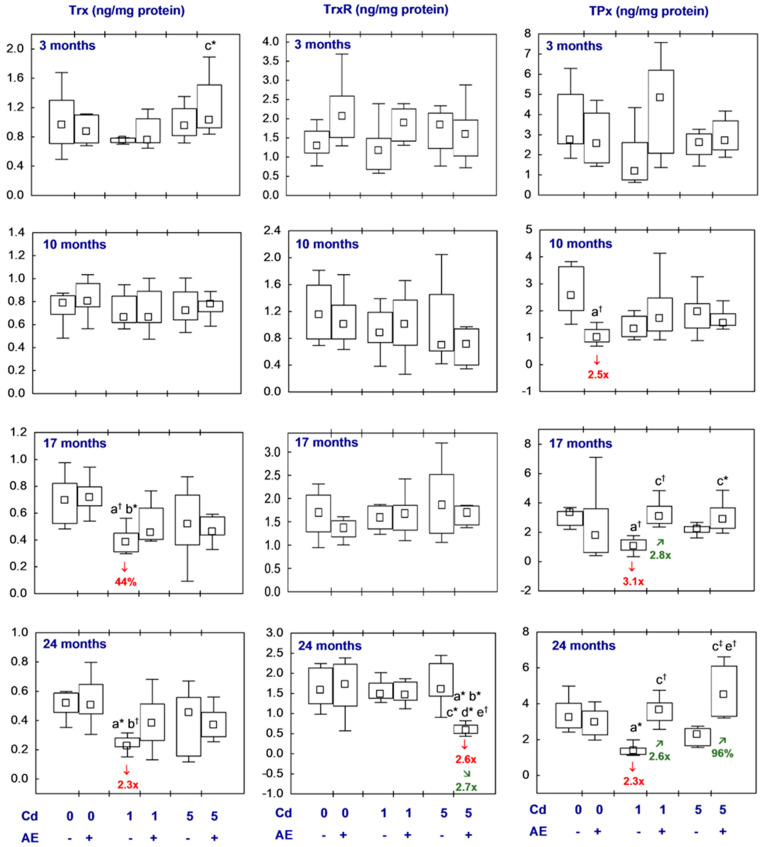
The concentrations of thioredoxin (Trx), thioredoxin reductase (TrxR), and thioredoxin peroxidase (TPx) in the brains of female rats treated with cadmium (Cd) (0, 1, or 5 mg Cd/kg diet) and/or 0.1% extract from *A. melanocarpa* L. berries (AE). Statistical differences from the: a—Control group, b—AE group, c—Cd_1_ group, d—Cd_1_ + AE group, and e—Cd_5_ group are marked as * *p* < 0.05, ^†^ *p* < 0.01, and ^‡^ *p* < 0.001. The differences in comparison to the control animals (↓, lower) and the appropriate Cd group (↗, higher and ↘, lower) are marked with the numerical values indicating a percentage difference or a fold of difference between the respective groups. The effect size (η^2^) for the differences in the concentration of Trx between the experimental groups was medium-to-large (0.130–0.300) and large for TrxR and TPx (0.330 and 0.290–0.640, respectively). Detailed data on the concentrations of Trx, TrxR, and TPx (including η^2^) are provided in Table S5.

**Figure 5:**
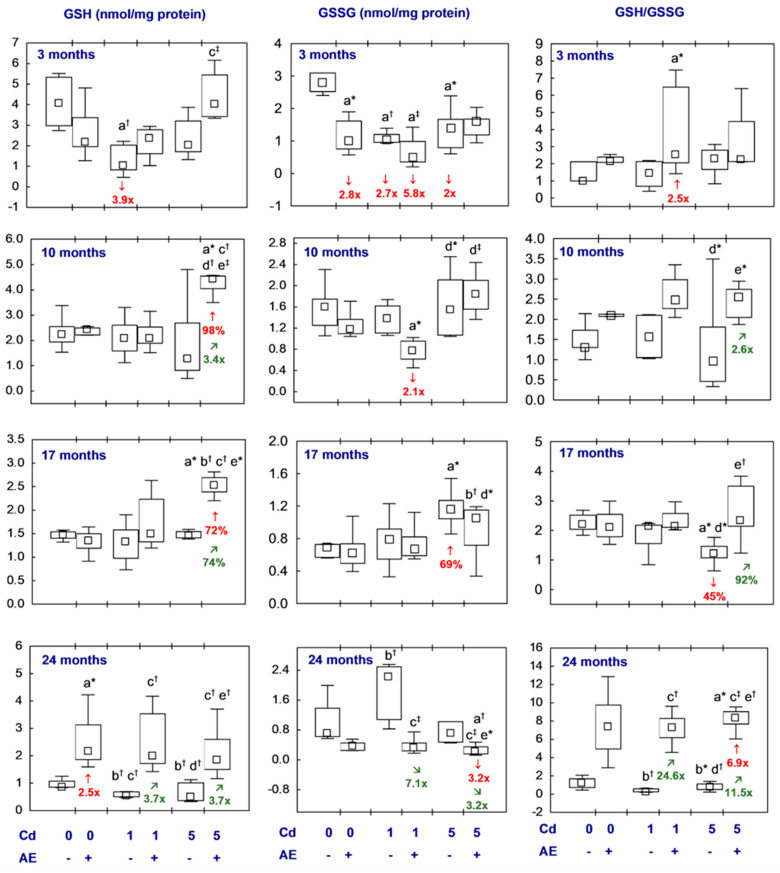
The concentrations of reduced glutathione (GSH) and oxidized glutathione (GSSG) and their ratio (GSH/GSSG) in the brains of female rats treated with cadmium (Cd) (0, 1, or 5 mg Cd/kg diet) and/or 0.1% extract from *A. melanocarpa* L. berries (AE). Statistical differences from the: a—Control group, b—AE group, c—Cd_1_ group, d—Cd_1_ + AE group, and e—Cd_5_ group are marked as * *p* < 0.05, ^†^ *p* < 0.01, and ^‡^ *p* < 0.001. The differences in comparison to the control animals (↑, higher and ↓, lower) and the appropriate Cd group (↗, higher and ↘, lower) are marked with the numerical values indicating a percentage difference or a fold of difference between the respective groups. The effect size (η^2^) for the differences in the concentrations of GSH and GSSG and the GSH/GSSG ratio between the experimental groups was large (0.361–0.773, 0.297–0.717, and 0.264–0.789, respectively). Detailed data on the parameters of glutathione homeostasis (including η^2^) are provided in Table S6.

**Figure 6:**
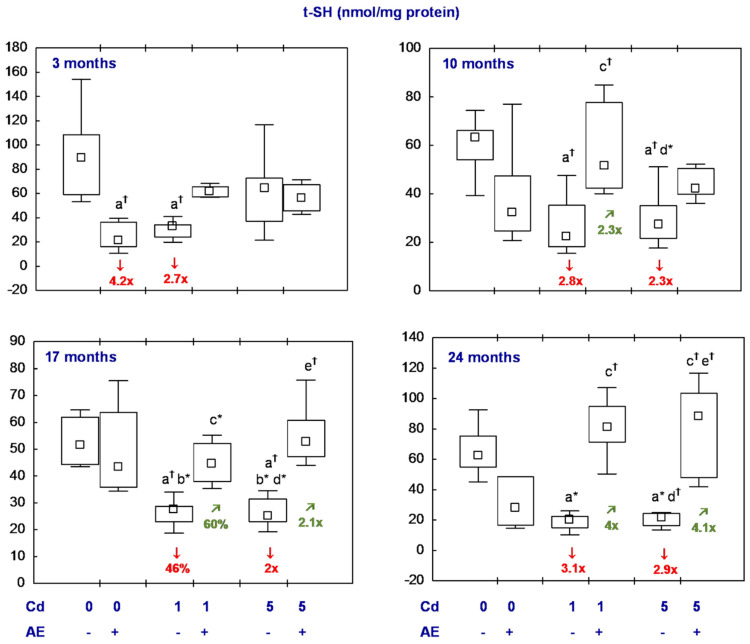
The concentration of total thiol groups (t-SH) in the brains of female rats treated with cadmium (Cd) (0, 1, or 5 mg Cd/kg diet) and/or 0.1% extract from *A. melanocarpa* L. berries (AE). Statistical differences from the: a—Control group, b—AE group, c—Cd_1_ group, d—Cd_1_ + AE group, and e—Cd_5_ group are marked as * *p* < 0.05 and ^†^ *p* < 0.01. The differences in comparison to the control animals (↓, lower) and the appropriate Cd group (↗, higher) are marked with the numerical values indicating a percentage difference or a fold of difference between the respective groups. The effect size (η^2^) for the differences in the concentration of t-SH between the experimental groups was large (0.430–0.650). Detailed data on the concentration of t-SH (including η^2^) are provided in Table S7.

**Figure 7:**
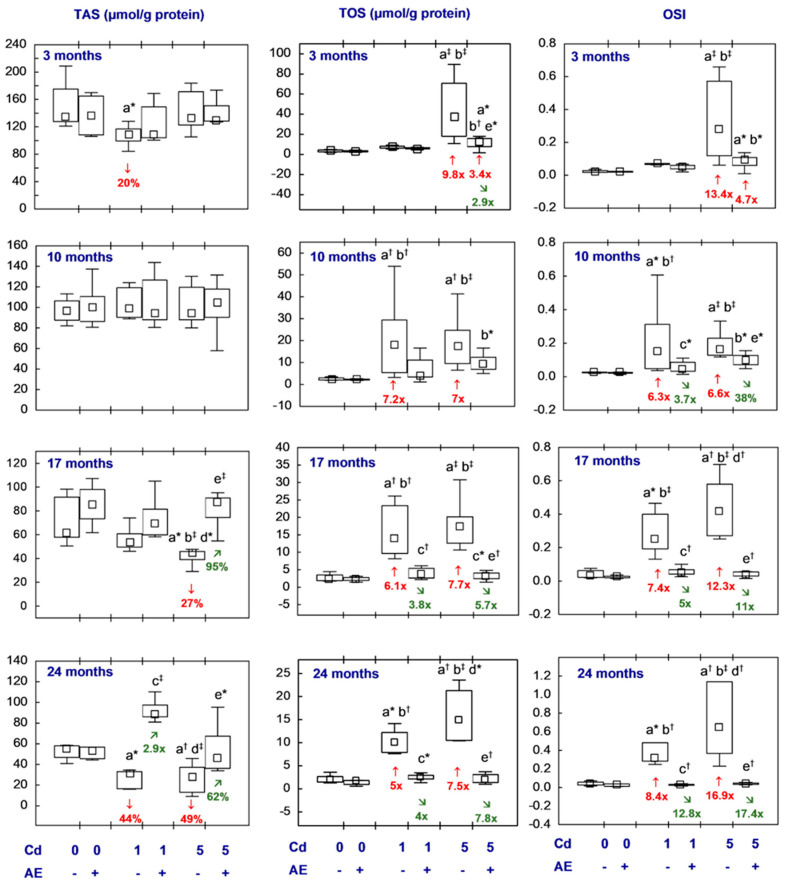
Total antioxidative status (TAS), total oxidative status (TOS), and oxidative stress index (OSI) in the brains of female rats treated with cadmium (Cd) (0, 1, or 5 mg Cd/kg diet) and/or 0.1% extract from *A. melanocarpa* L. berries (AE). Statistical differences from the: a—Control group, b—AE group, c—Cd_1_ group, d—Cd_1_ + AE group, and e—Cd_5_ group are marked as * *p* < 0.05, ^†^ *p* < 0.01, and ^‡^ *p* < 0.001. The differences in comparison to the control animals (↑, higher and ↓, lower) and to the appropriate Cd group (↗, higher and ↘, lower) are marked with the numerical values indicating a percentage difference or a fold of difference between the respective groups. The effect size (η^2^) for the differences in the values of TAS, TOS, and OSI between the experimental groups was large (0.198–0.697, 0.626–0.747, and 0.656–0.735, respectively). Detailed data on TAS and TOS (including η^2^) are provided in Table S8.

**Figure 8:**
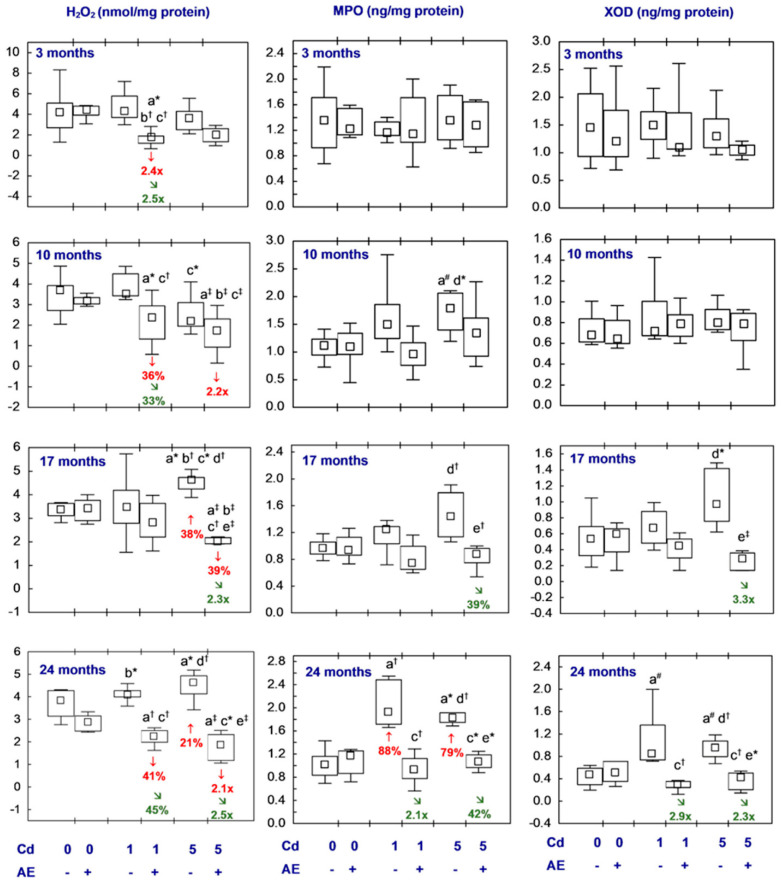
The concentrations of hydrogen peroxide (H_2_O_2_), xanthine oxidase (XOD), and myeloperoxidase (MPO) in the brains of female rats treated with cadmium (Cd) (0, 1, or 5 mg Cd/kg diet) and/or 0.1% extract from *A. melanocarpa* L. berries (AE). Statistical differences from the: a—Control group, b—AE group, c—Cd_1_ group, d—Cd_1_ + AE group, and e—Cd_5_ group are marked as * *p* < 0.05, ^†^ *p* < 0.01, ^‡^ *p* < 0.001, and ^#^ *p* = 0.05–0.08. The differences in comparison to the control animals (↑, increase and ↓, lower) and the appropriate Cd group (↘, lower) are marked with the numerical values indicating a percentage difference or a fold of difference between the respective groups. The effect size (η^2^) for the differences in the concentrations of H_2_O_2_, XOD, and MPO between the experimental groups was large (0.380–0.750, 0.300–0.600, and 0.340–0.580, respectively). Detailed data on the concentrations of H_2_O_2_, XOD, and MPO (including η^2^) are provided in Table S9.

**Figure 9:**
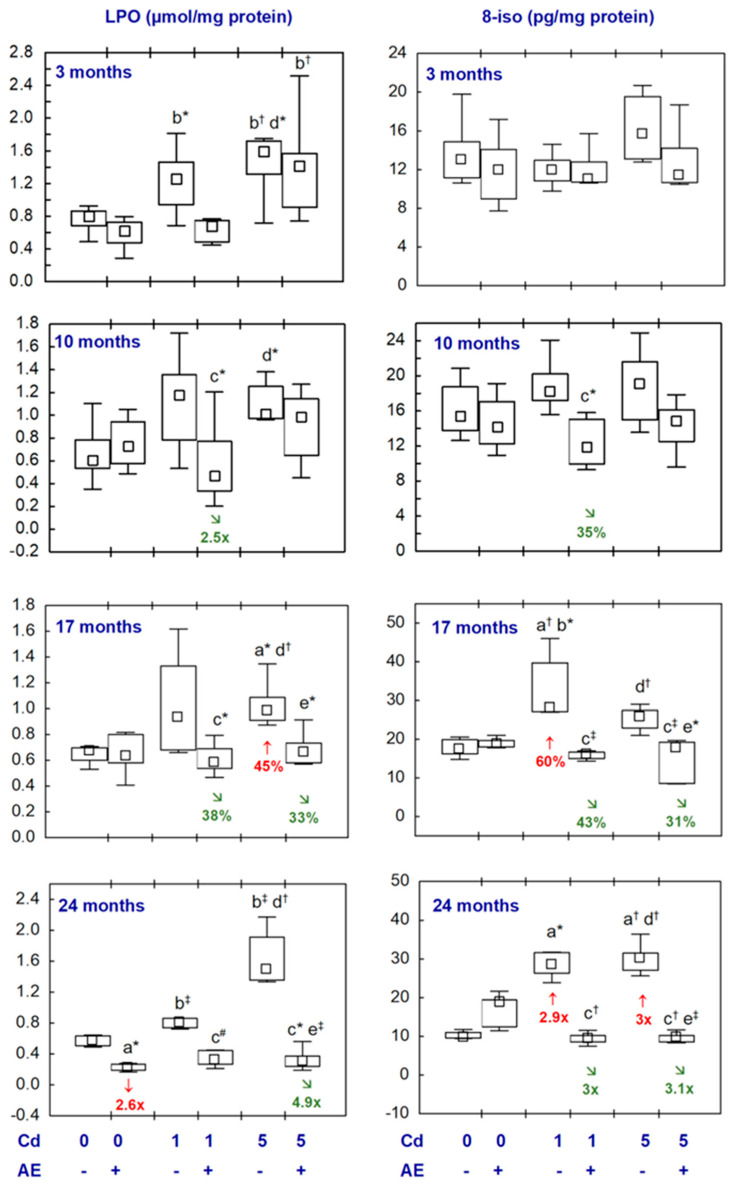
The concentrations of lipid peroxides (LPO) and 8-isoprostane (8-iso) in the brains of female rats treated with cadmium (Cd) (0, 1, or 5 mg Cd/kg diet) and/or 0.1% extract from *A. melanocarpa* L. berries (AE). Statistical differences from the: a—Control group, b—AE group, c—Cd_1_ group, d—Cd_1_ + AE group, and e—Cd_5_ group are marked as * *p* < 0.05, ^†^ *p* < 0.01, ^‡^ *p* < 0.001, and ^#^ *p* = 0.05. The differences in comparison to the control animals (↑, higher and ↓, lower) and the appropriate Cd group (↘, lower) are marked with the numerical values indicating a percentage difference or a fold of difference between the respective groups. The effect size (η^2^) for the differences in the concentrations of LPO and 8-iso between the experimental groups was large (0.240–0.820 and 0.210–0.770, respectively). Detailed data on the concentrations of LPO and 8-iso (including η^2^) are provided in Table S10.

**Figure 10:**
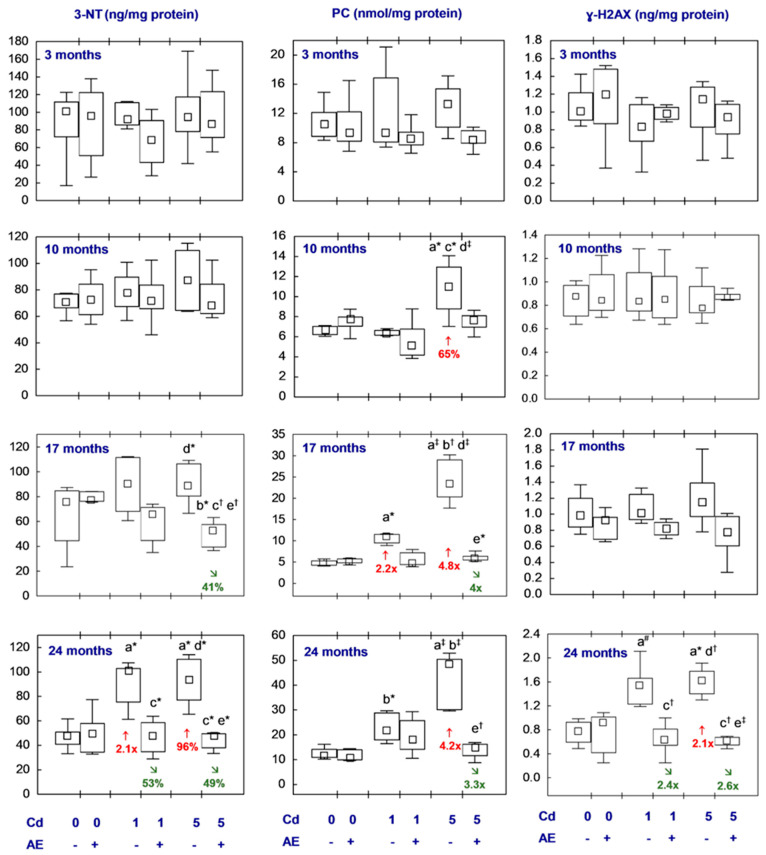
The concentrations of 3-nitrotyrosine (3-NT), protein carbonyl groups (PC), and ɣ-H2A histone family member X (ɣ-H2AX) in the brains of female rats treated with cadmium (Cd) (0, 1, or 5 mg Cd/kg diet) and/or 0.1% extract from *A. melanocarpa* L. berries (AE). Statistical differences from the: a—Control group, b—AE group, c—Cd_1_ group, d—Cd_1_ + AE group, and e—Cd_5_ group are marked as * *p* < 0.05, ^†^ *p* < 0.01, ^‡^ *p* < 0.001, and ^#^ *p* = 0.05. The differences in comparison to the control animals (↑, higher) and the appropriate Cd group (↘, lower) are marked with the numerical values indicating a percentage difference or a fold of difference between the respective groups. The effect size (η^2^) for the differences in the concentrations of 3-NT, PC, and ɣ-H2AX between the experimental groups was large (0.420–0.520, 0.390–0.650, and 0.640, respectively). Detailed data on the concentrations of 3-NT, PC, and ɣ-H2AX (including η^2^) are provided in Table S11.

All statistical analyses were conducted in the Statistica 13.3 package (StatSoft, Tulsa, OK, USA). The data distribution was verified using the Shapiro–Wilk test. As a normal distribution was not revealed in any of the groups, the non-parametric Kruskal–Wallis test was used to determine if the differences between the groups were statistically significant (the level of significance was stated as 0.05). The results of the measurements of particular parameters are presented in Figure 2, Figure 3, Figure 4, Figure 5, Figure 6, Figure 7, Figure 8, Figure 9 and Figure 10 as a median and minimum and maximum values for 8 animals, except for 7 females in three groups (the AE, Cd_1_, and Cd_5_ groups) after 24 months. Moreover, quantitative differences (percentage difference or fold of difference) in particular variables in the rats administered cadmium and/or AE compared to the control animals, as well as between the Cd_1_ + AE and Cd_5_ + AE groups and the respective groups that did not receive AE (the Cd_1_ and Cd_5_ groups) are marked in Figure 2, Figure 3, Figure 4, Figure 5, Figure 6, Figure 7, Figure 8, Figure 9 and Figure 10. In cases when the value of a parameter in one group was higher than its value in another group by more than 100% or was lower by 50% or more, a factor of difference between these two groups was provided. To evaluate the effect size for the differences (the strength of the difference) in the values of particular parameters between the experimental groups at any time point, eta squared (η^2^) was calculated and the results are summarized in the appropriate figures (Figure 2, Figure 3, Figure 4, Figure 5, Figure 6, Figure 7, Figure 8, Figure 9 and Figure 10) and presented in detail in tables provided as the Supplementary Materials. When the value of η^2^ is ≤0.01 the effect size is described as small, for the values of η^2^ ≥ 0.14 the effect is large, whereas in the case of 0.01 < η^2^ < 0.14, it is medium.

A linear regression analysis demonstrating the β coefficient and R^2^ was conducted to predict mutual dependencies between the values of the examined parameters, as well as to show if there was a dependence between these variables and the cadmium concentration in the brain, blood, and urine (previously published [39]). The significance level was stated as *p* < 0.05. The β coefficient shows the percentage of change of the dependent variable for a one-unit change of the independent variable, whereas R^2^ represents how much the percentage of one variable can be explained by the variability of the other variable (the effect size). The effect size for the regression analysis is small, medium, and large when R^2^ ≤ 0.02, 0.02 < R^2^ < 0.26, and R^2^ ≥ 0.26, respectively.

## 3. Results

### 3.1. Macroscopic Picture and Weight of the Brain

The brains of the control rats showed a normal macroscopic picture. There were no signs of cerebral oedema, congestion, or bleeding. In the animals fed with the 1 and 5 mg Cd/kg diet and/or supplemented with AE, this organ macroscopic picture did not differ compared to the control animals.

The brain median absolute weight in the control animals was 1.7212 g (1.3799–1.9432 g) after 3 months of the experiment and 2.0551 g (1.8462–2.0964 g) after 24 months of its duration. This organ’s relative weight in the control animals after 3 and 24 months reached 0.5519 g/100 g b.w. (0.3988–0.6035 g/100 g b.w.) and 0.3133 g/100 g b.w. (0.2669–0.3813 g/100 g b.w.), respectively (Table S2). There were no differences in the absolute weight or the relative weight of the brain between the study groups throughout the experiment (Table S2).

### 3.2. Antioxidative Barrier of the Brain

#### 3.2.1. Antioxidative Enzymes (SOD, CAT, GPx, and GR)

The supplementation with AE alone (AE group) did not influence the activities of SOD, CAT, GPx, or GR in the brain, except for the activity of GR, which was lower than in the control animals after 17 and 24 months (3.9- and 2.7-fold, respectively) (Figure 2 and Figure 3, Tables S3 and S4).

The brain activity of SOD in the females maintained on the 1 mg Cd/kg diet alone (the Cd_1_ group) and together with the supplementation with AE (the Cd_1_ + AE group) for 3–24 months was within the range of values determined in the control animals (Figure 2, Table S3). In the Cd_5_ group, the activity of this enzyme after 17 and 24 months was lower (by 33% and 2-fold, respectively), whereas a shorter exposure to cadmium did not influence this parameter (Figure 2, Table S3). In the females co-administered cadmium in the diet at the concentration of 5 mg/kg and AE, the activity of SOD after 10, 17, and 24 months was higher (by 36–90%) than in the corresponding group receiving only cadmium. No difference in the activity of SOD between the Cd_5_ + AE and control groups throughout the study was noted (Figure 2, Table S3).

There was a decrease (2.1-fold) in CAT activity in the females intoxicated with the 1 mg Cd/kg diet (the Cd_1_ group) for 3 months and a tendency to decrease in this parameter after 10 and 24 months (*p* = 0.07 and *p* = 0.08, respectively) (Figure 2, Table S3). In the Cd_1_ + AE group, this enzyme activity throughout the whole experiment did not differ from the control animals, and after 3, 10, and 24 months it was higher than in the Cd_1_ group (from 2.2- to 2.6-fold) (Figure 2, Table S3). The rats receiving the diet containing cadmium at the higher concentration (the Cd_5_ group) for 17 and 24 months had lower CAT activity in the brain than the control animals (2.3- and 2.7-fold, respectively), but after a shorter treatment, the activity was unaffected (Figure 2, Table S3). In the females co-administered the 5 mg Cd/kg diet and AE, the activity of CAT throughout the experiment exceeded (from 2.1- to 2.7-fold) the activity determined in the Cd_5_ group but was within the range of the proper values assayed in the control rats (Figure 2, Table S3).

The brain activity of GPx in the Cd_1_ group was unchanged, whereas GR activity was decreased after 10 and 17 months (by 35% and 2.4-fold, respectively) and unaffected after 3 and 24 months (Figure 3, Table S4). The rats treated with the 5 mg Cd/kg diet had lower activity of GPx after 10–24 months (from 2- to 6.8-fold) and higher GR activity after 24 months (by 42%) (Figure 3, Table S4). In the rats supplemented with AE and exposure to 1 and 5 mg Cd/kg diet, the activities of both enzymes were within the proper values assayed in the control females, except for a lower activity of GR after 17 months (2.3- and 2.4-fold, respectively) (Figure 3, Table S4). After 17 and 24 months, the brain activity of GPx in the Cd_5_ + AE group exceeded (2.3- and 2.6-fold, respectively) the activity of this enzyme determined in the Cd_5_ group, while GR activity was lower in the former group (2.3-fold and by 46%, respectively) (Figure 3, Table S4).

There were no differences in the activities of the assayed antioxidative enzymes in the brain between the Cd_1_ and Cd_5_ groups and between the Cd_1_ + AE and Cd_5_ + AE groups, except for a lower activity of SOD after 17 and 24 months (by 32% and 2-fold, respectively) and a higher (2.3-fold) activity of GR after 17 months of the moderate exposure to cadmium (the Cd_5_ group) than the low-level treatment (the Cd_1_ group) (Figure 2 and Figure 3, Tables S3 and S4).

#### 3.2.2. Trx-Dependent Antioxidative System

The investigated indices of the Trx-dependent antioxidative system (Trx, TrxR, and TPx) were not influenced by up to 24 months application of AE alone. The only exception was the concentration of TPx after 10 months, which was lower (2.5-fold) than in the control females (Figure 4, Table S5).

The females fed with the 1 mg Cd/kg diet for 17 and 24 months had lower Trx concentrations (by 44% and 2.3-fold, respectively) in the brain, whereas the shorter low-level intoxication with this xenobiotic and the administration of the diet containing cadmium at the higher concentration throughout the whole experiment did not influence this parameter (Figure 4, Table S5). The Trx concentration in the Cd_1_ + AE and Cd_5_ + AE groups at all time points did not differ compared to the control animals and the respective groups treated with cadmium alone (Figure 4, Table S5).

The exposure to cadmium at both concentrations in the diet, alone and together with the supplementation with AE, for the whole experiment had no impact on the concentration of TrxR, except for its lower value after 24 months in the Cd_5_ + AE group than in the control and the animals treated with cadmium alone (2.6- and 2.7-fold, respectively) (Figure 4, Table S5).

The rats fed with the diet containing cadmium at the lower of the investigated concentrations (the Cd_1_ group) had a decreased TPx concentration after 17 and 24 months (3.1- and 2.3-fold, respectively) compared to the control animals, while the higher exposure (the Cd_5_ group) did not influence the value of this parameter throughout the whole experiment (Figure 4, Table S5). In the animals from the Cd_1_ + AE group, the brain TPx concentration reached higher values after 17 and 24 months (2.8- and 2.6-fold, respectively) than in the rats fed the 1 mg Cd/kg diet, but it did not differ from the control animals (Figure 4, Table S5). In the females co-administered the 5 mg Cd/kg diet and AE for 24 months, the brain TPx concentration was higher (by 96%) than in the females administered cadmium alone. The concentration of this parameter in the rats supplemented with AE under the low-level and moderate treatment with cadmium was within the values noted in the control females (Figure 4, Table S5).

The brain concentrations of Trx, TrxR, and TPx did not differ between the Cd_1_ and Cd_5_ groups or between the Cd_1_ + AE and Cd_5_ + AE groups, except for a lower (2.4-fold) value of TrxR after 24 months of supplementation with AE at the moderate exposure to cadmium than under the low-level treatment (Figure 4, Table S5).

#### 3.2.3. Glutathione Homeostasis

The brain concentrations of GSH and GSSG and their ratio in the AE group were within the range of values noted in the control females, except for a higher (2.5-fold) concentration of GSH after the longest course of the experiment and a lower (2.8-fold) concentration of GSSG after its shortest duration (Figure 5, Table S6).

The low-level and moderate exposure to cadmium had only a temporary effect on GSH and GSSG concentrations in the brain and their ratio (Figure 5, Table S6). A decrease in the levels of GSH and GSSG in the Cd_1_ group (3.9- and 2.7-fold, respectively) and that of GSSG in the Cd_5_ group (2-fold) was noted after the shortest experimental period. Moreover, after 17 months of moderate exposure to cadmium the brain GSSG concentration was higher (by 69%) and the GSH/GSSG ratio was lower (by 45%) than in the control animals (Figure 5, Table S6).

The concentrations of GSH and GSSG and their ratio in the females co-administered AE and the 1 mg Cd/kg diet did not differ from the control animals, except for a lower GSSG concentration after 3 and 10 months (5.8- and 2.1-fold, respectively) and a higher ratio of GSH/GSSG after the shortest duration of the experiment (2.5-fold). These parameters in the Cd_1_ + AE group did not deviate from the values noted in the Cd_1_ group, with the exception of a higher GSH concentration and the ratio of GSH/GSSG (3.7- and 24.6-fold, respectively) and a lower GSSG concentration (7.1-fold) after 24 months (Figure 5, Table S6). In the rats supplemented with AE at the moderate cadmium exposure (the Cd_5_ group), the GSH concentration and GSH/GSSG ratio during the experiment, except for its 3-month duration, were elevated (from 74% to 11.5-fold) compared to the Cd_5_ group. Moreover, the concentration of GSH in these animals after 10 and 17 months and the GSH/GSSG ratio after the longest co-administration of AE and the 5 mg Cd/kg diet were higher than in the control females (by 98%, by 72%, and 6.9-fold, respectively), whereas the GSSG concentration after the 2-year experiment duration was lower compared to the control group (3.2-fold) (Figure 5, Table S6).

The brain concentrations of GSH and GSSG and their ratio did not differ between the Cd_1_ group and the Cd_5_ group, or between the Cd_1_ + AE group and the Cd_5_ + AE group. The only exception was a higher GSH concentration after 10 months (2.1-fold) and a higher GSSG concentration after 10 and 17 months (2.4-fold and by 58%, respectively) in the Cd_5_ + AE group than in the Cd_1_ + AE group (Figure 5, Table S6).

#### 3.2.4. t-SH

The concentration of t-SH in the females receiving AE alone did not differ from the control group, except for its lower (4.2-fold) value after the shortest administration of the extract (Figure 6, Table S7). In the Cd_1_ and Cd_5_ groups, the t-SH concentration was lower than in the control animals throughout the whole experiment (from 46% to 3.1-fold) with one exception. The concentration was unaffected due to the shortest, moderate treatment with cadmium. In the groups supplemented with AE under exposure to this harmful element, the concentration of t-SH was within the range of the control group throughout the study, and in the Cd_1_ + AE group, it reached a higher value than in the Cd_1_ group after 10, 17, and 24 months (2.3-fold, by 60%, and 4-fold, respectively), whereas in the animals co-administered the 5 mg Cd/kg diet and AE for 17 and 24 months it was higher (2.1- and 4.1-fold, respectively) than in these receiving this diet alone (Figure 6, Table S7).

No differences were noted throughout the whole experiment in the concentration of t-SH in the brain between the Cd_1_ and Cd_5_ groups and between the Cd_1_ + AE and Cd_5_ + AE groups (Figure 6, Table S7).

### 3.3. TAS, TOS, and OSI in the Brain

In the females receiving AE alone, the brain TOS, TAS, and OSI reached values comparable to those in the control rats (Figure 7, Table S8).

In the Cd_1_ and Cd_5_ groups, TAS was decreased, while TOS and OSI were higher than in the control females. The impact of cadmium depended on both the intensity of exposure and its duration. TAS was decreased due to the shortest low-level exposure to cadmium (by 20%) and the 17-month moderate treatment (by 27%), as well as after 24 months regardless of the level of intoxication (by 44% and 49%, respectively). TOS and OSI were increased at each time point of the low-level and moderate exposure (5–7.2 and 6.3–8.4 times in the Cd_1_ group, respectively, and 7–9.8 and 6.6–16.9 times in the Cd_5_ group, respectively), except for the shortest low-level treatment (Figure 7, Table S8).

In the females subjected to AE supplementation under the treatment with cadmium (the Cd_1_ + AE and Cd_5_ + AE groups), TOS, TAS, and OSI were within the values noted in the control animals, with the exception of higher TOS and OSI after the shortest co-administration of the 5 mg Cd/kg diet and AE (3.4- and 4.7-fold, respectively). In the animals of the Cd_5_ + AE group, TAS after 17 months of the study exceeded (by 95%) the value determined in the Cd_5_ group, whereas this parameter after the 24-month supplementation with AE under exposure to cadmium (the Cd_1_ + AE and Cd_5_ + AE groups) exceeded the values noted in the respective groups treated with cadmium alone (2.9-fold and by 62%, respectively). For the co-administration of cadmium and AE, TOS and OSI, at numerous time points, reached values lower (from 38% to 17.4-fold) than in the case of no supplementation with this extract during the treatment with cadmium (Figure 7, Table S8).

Throughout the study, no differences in TAS, TOS, or OSI between the Cd_1_ and Cd_5_ groups or the Cd_1_ + AE and Cd_5_ + AE groups were noted (Figure 7, Table S8).

### 3.4. Concentrations of H_2_O_2_, MPO, and XOD in the Brain

The brain concentrations of H_2_O_2_, MPO, and XOD in the female rats administered only AE did not differ compared to the control ones (Figure 8, Table S9).

Feeding the diet containing 1 mg Cd/kg did not influence the H_2_O_2_ concentration in the nervous tissue of brain origin (Figure 8, Table S9). In the females supplemented with AE under the low-level treatment with cadmium (the Cd_1_ + AE group), the value of this parameter at all time points, except for 17 months, was lower compared to the control animals (from 36% to 2.4-fold), as well as the group treated with cadmium alone (from 33% to 2.5-fold) (Figure 8, Table S9). The H_2_O_2_ concentration after 17 and 24 months of the moderate treatment with cadmium alone was higher (by 38% and 21%) than in the control rats (Figure 8, Table S9). Moreover, the concentration of this compound in the animals co-administered the 5 mg Cd/kg diet and AE for 10, 17, and 24 months reached lower values compared to the control animals (by 39% to 2.2-fold) and after the 17- and 24-month duration of the study they were also lower compared to the females that were not supplemented with AE during the moderate treatment with cadmium alone (the Cd_5_ group) (2.3- and 2.5-fold, respectively) (Figure 8, Table S9).

The concentration of MPO in the females maintained on the 5 mg Cd/kg diet tended (*p* = 0.05) to increase after 10 months but after the longest low-level and moderate treatment it was increased (by 88% in the Cd_1_ group and 79% in the Cd_5_ group, respectively) (Figure 8, Table S9). In the groups supplemented with AE under the treatment with cadmium (the Cd_1_ + AE and Cd_5_ + AE groups), the concentration of this prooxidant throughout the study was within the range of values noted in the control females, and after the longest duration of the study, it was lower than in the respective groups receiving only cadmium (2.1-fold and by 42%, respectively) (Figure 8, Table S9).

The administration of cadmium at both concentrations in the diet and AE alone and together for 3–17 months did not influence the brain concentration of XOD, but after 17 months this parameter in the Cd_5_ + AE group was lower (3.3-fold) than in the case of the moderate treatment with cadmium alone. At the end of the 24-month study, the concentration of XOD under low and moderate intoxication with cadmium showed a growing tendency (*p* = 0.06 and 0.08, respectively), whereas, in the case of simultaneous supplementation with AE, the concentration did not differ from the control animals and was lower (2.9- and 2.3-fold, respectively) than in the appropriate animals receiving cadmium alone (Figure 8, Table S9).

The brain concentrations of H_2_O_2_, MPO, and XOD did not differ between the low-level and moderate exposure to cadmium (the Cd_1_ group versus the Cd_5_ group) or between the groups supplemented with AE during the treatment with this toxic element (the Cd_1_ + AE group versus the Cd_5_ + AE group), except for the concentration of H_2_O_2_ in the animals fed the 5 mg Cd/kg diet alone, which after 10 months was lower and after 17 months was higher than with the low-level intoxication (the Cd_5_ group versus the Cd_1_ group) (by 37% and 32%, respectively) (Figure 8, Table S9).

### 3.5. Biomarkers of Oxidative Damage to Lipids, Proteins, and DNA in the Brain

The concentrations of all biomarkers of lipid peroxidation (LPO and 8-iso), and oxidative modifications of proteins (3-NT and PC) and DNA (ɣ-H2AX) in the nervous tissues of brain origin of the animals receiving AE alone were comparable to these determined in the control females, with one exception only, i.e., a lower (2.6-fold) LPO concentration at the end of the 24-month study (Figure 9 and Figure 10, Tables S10 and S11).

The low-level exposure to cadmium via diet did not influence the LPO concentration in the brain (Figure 9, Table S10). The concentration of this marker of lipid peroxidation in the animals supplemented with AE under the low-level treatment with cadmium (the Cd_1_ + AE group) throughout the study did not differ from the value of this parameter determined in control ones, but after 10 and 17 months it reached lower values compared to the group administered the 1 mg Cd/kg diet alone (2.5-fold and by 38%, respectively) and tended (*p* = 0.05) to be lower after 24 months (Figure 9, Table S10). The brain concentration of LPO after the 17-month feeding with the 5 mg Cd/kg diet alone was higher (by 45%) relative to the control females (Figure 9, Table S10). The rats receiving AE supplementation under exposure to the 5 mg Cd/kg diet for 17 and 24 months had lower concentrations of LPO (by 33% and 4.9-fold, respectively) than the animals administered only cadmium, and the value of this parameter was comparable to the concentration assayed in the control animals (Figure 9, Table S10).

The 17- and 24-month feeding with the 1 mg Cd/kg diet enhanced (by 60% and 2.9-fold, respectively) the concentration of 8-iso, whereas the shorter exposure had no impact on this parameter compared to the control group (Figure 9, Table S10). The concentration of 8-iso in the Cd_1_ + AE group throughout the study ranged within the values noted in the control animals, and after 10, 17, and 24 months it was lower compared to the females that did not receive the extract under the low-level cadmium intoxication (by 35%, by 43%, and 3-fold, respectively). The females subjected to the 24-month feeding with the 5 mg Cd/kg diet had a 3-fold higher concentration of 8-iso than the control animals, whereas simultaneous supplementation with AE allowed them to maintain the concentration of this biomarker of lipid peroxidation at the level of the control, and the value of this parameter was 3.1-fold lower than in the group treated with cadmium alone. Moreover, the brain concentration of 8-iso in the rats from the Cd_5_ + AE group was lower (by 31%) compared to the respective group treated with cadmium alone after the 17-month duration of the study (Figure 9, Table S10).

The 24-month low-level and moderate treatment with cadmium (the Cd_1_ and Cd_5_ groups) led to an elevation in 3-NT concentration in the nervous tissue of brain origin (2.1-fold and by 96%, respectively), whereas the shorter exposure had no impact on this parameter as compared to the control group (Figure 10, Table S11). In the females supplemented with AE under the 17- and 24-month moderate exposure to this toxic element, as well as under the 24-month low-level cadmium intoxication, the concentration of 3-NT was lower than in the respective group treated with Cd alone (by 41%, 49%, and 53%, respectively), and it was comparable to the control group. Feeding with the diet containing 1 mg Cd/kg for 17 months and 5 mg Cd/kg for 10, 17, and 24 months increased the PC concentration in the brain (2.2-fold, by 65%, 4.8-fold, and 4.2-fold, respectively) compared to the control group. The brain concentration of this parameter in the animals co-administered the 5 mg Cd/kg diet and AE for 17 and 24 months reached lower (4- and 3.3-fold, respectively) values compared to the Cd_5_ group. The concentration of PC in the rats supplemented with AE under the low-level and moderate cadmium treatment at each time point ranged within the values determined in the control animals (Figure 10, Table S11).

The administration of cadmium and AE alone or together for 3–17 months resulted in no differences in the concentration of ɣ-H2AX in the brain between the experimental groups (Figure 10, Table S11). In the rats maintained on the diet containing 1 mg Cd/kg for 24 months, a tendency (*p* = 0.05) for a higher value of this marker of oxidative damage to DNA was noted, whereas the long-term moderate treatment with this toxic heavy metal led to an increase in the concentration of this parameter (2.1-fold) in comparison to the value of the control group. The brain concentration of ɣ-H2AX after the 24-month co-administration of AE and the 1 or 5 mg Cd/kg diet (Cd_1_ + AE and Cd_5_ + AE groups) was lower (2.4- and 2.6-fold, respectively) than in the respective groups of animals that received cadmium alone and was comparable to its level in the control group (Figure 10, Table S11).

The concentrations of the measured indices of lipid peroxidation and oxidative damage to proteins and DNA showed no differences among the Cd_1_ and Cd_5_ groups, as well as the Cd_1_ + AE and Cd_5_ + AE groups, with only one exception, i.e., the higher (by 72%) PC concentration in the Cd_5_ group as compared to the Cd_1_ group after 10 months (Figure 9 and Figure 10, Tables S10 and S11).

### 3.6. Relationships between the Examined Parameters in the Brain

In the animals that did not receive AE supplementation (alone and under the treatment with cadmium), positive correlations occurred between TAS of the brain and CAT activity, GPx activity, TPx concentration, GSH concentration, t-SH concentration, and the ratio of GSH/GSSG (Table 1). TAS negatively correlated with XOD, MPO, LPO, 8-iso, 3-NT, PC, and ɣ-H2AX concentrations, whereas between TOS and the activity of GR and the concentration of H_2_O_2_, positive correlations occurred (Table 1). Negative relationships were noted between the TOS of the brain and the concentrations of t-SH and TPx, as well as the activities of antioxidative enzymes such as CAT, SOD, and GPx. Moreover, the brain GSH concentration showed a tendency to correlate negatively (*p* = 0.05) with TOS (Table 1). The brain OSI negatively correlated with t-SH concentration and the activities of CAT and GPx and positively with the concentrations of MPO, LPO, and 8-iso (Table 1). Negative dependencies were noted between the brain TAS and TOS (β =—0.740, *p* < 0.001, R^2^ = 0.521) and OSI (β =—0.760, *p* < 0.001, R^2^ = 0.554), whereas in this organ, TOS positively correlated with OSI (β = 0.780, *p* < 0.001, R^2^ = 0.589).

In the females administered AE (alone and under the treatment with cadmium), positive relationships between TAS and the activity of GR and the concentration of PC and a negative dependency between TOS and the concentration of H_2_O_2_ were noted (Table 1). A positive dependence occurred between the brain OSI in these animals and the concentration of TPx, whereas the concentrations of H_2_O_2_ and ɣ-H2AX correlated negatively with OSI (Table 1). Moreover, positive dependencies occurred between TAS and TOS of the brain (β = 0.510, *p* < 0.05, R^2^ = 0.225) and between TOS and OSI (β = 0.566, *p* < 0.01, R^2^ = 0.288).

The effect size (R^2^) for the above-described relationships noted in both the female rats that were not supplemented with AE and the animals supplemented with the extract was medium-to-large (Table 1).

### 3.7. Relationships between Investigated Parameters and Cadmium Concentration in the Brain, Blood, and Urine

In the animals that were not treated with AE, negative correlations were noted between the brain activities of CAT, SOD, and GPx and the cadmium concentration in the brain, blood, and urine (Table 2). The cadmium concentration in the brain, blood, and urine positively correlated with the TOS, OSI, and MPO concentration, as well as the levels of biomarkers of lipid peroxidation (LPO and 8-iso) and oxidative modifications of proteins (3-NT and PC) and DNA (ɣ-H2AX) in the brain, except for a lack of a correlation between the concentrations of 8-iso and cadmium in the brain and the brain concentration of ɣ-H2AX and the cadmium concentration in the urine (Table 2). Moreover, a positive dependence occurred between the brain concentrations of XOD and cadmium (Table 2).

In the rats supplemented with AE, there were positive dependencies between the brain concentrations of GSH and LPO, TOS, and OSI and the cadmium concentration in the brain, blood, and urine, whereas, between these concentrations of cadmium and the H_2_O_2_ concentration in the brain, a negative dependence occurred (Table 2). A positive correlation was noted between the brain GR activity and the cadmium concentration (Table 2). Moreover, negative dependencies occurred between the 8-iso and cadmium concentrations in the nervous tissue of brain origin, as well as the concentrations of TrxR and ɣ-H2AX in the nervous tissue and the cadmium concentration in the blood (Table 2). Positive relationships were noted between the GSSG concentration in the brain and the cadmium concentration in the blood and urine (Table 2).

The effect size (R^2^) for the relationships between markers of the oxidative–antioxidative status in the brain and the cadmium concentration in the brain, blood, and urine in the rats not supplemented with AE was medium-to-large, whereas, in the animals receiving the supplementation, it was medium (Table 2).

## 4. Discussion

This research is the first investigation pointing out, in the experimental female rat model of the exposure of the general population to cadmium currently occurring in developed countries worldwide, that chronic low-to-moderate intoxication with this xenobiotic destroyed the balance between the processes of oxidation and reduction, resulting in the development of oxidative stress and oxidative injury of lipids, proteins, and DNA in the brain, whereas the co-administration of AE can attenuate these effects or even prevent them. Since oxidative stress in the brain, regardless of its cause, leads to impairment in the functioning of the nervous system cells [5,15,16,17,18,19,20,21,22,23,24,25,26,27,28,29,30,31,32,33,34], the results of the present study indicated that long-lasting, even low-level, exposure to cadmium can pose a risk of failure of the nervous system by disorder of the balance between the processes of oxidation and reduction in the brain and that the administration of a powerful antioxidant such as AE during intoxication with this trace element can alleviate its injurious influence on the nervous tissue. Thus, this study provided important data not only on the risk of the damaging impact of cadmium under low-to-moderate chronic dietary exposure lasting for up to the entire lifetime but also on the possibility of effective protection against this impact with the use of foods characterized by high nutritional value, such as aronia-based products.

The brain is a very important organ in the body that forms, together with the spinal cord, the central nervous system. This organ is protected to some extent from the destroying influence of harmful factors, including xenobiotics, by the blood–brain barrier [5,27,58]. This structure, built from endothelial cells, regulates the transport of both essential and toxic substances between the brain and the blood [27,58]. However, many factors, such as but not limited to diseases of the nervous system, some drugs, and toxic heavy metals, like cadmium, can damage this barrier and increase its permeability to toxic substances [5,57]. The transportation of cadmium across the intact blood–brain barrier is low and thus the amount of this toxic heavy metal in the brain is significantly lower than in tissues well supplied with blood such as the liver and kidneys [39,40]. However, as it was revealed by our research team in the rat model of human exposure to cadmium during the lifetime, this element, even at low-level intoxication, entered the brain, where it accumulated and exerted toxic action [33,39,40]. It needs to be emphasized that cadmium penetration via the blood–brain barrier may be facilitated as a result of its unfavourable impact on this barrier [5,27,34].

This study showed that the low-to-moderate-level long-lasting treatment of female rats with cadmium, covering almost the whole lifetime, led to a state in which the processes of oxidation dominated over the processes of reduction, which resulted in oxidative modifications of the basic macromolecules in the nervous system cells, i.e., proteins, lipids, and nucleic acids. Although at particular time points, no differences (with only a few exceptions) in the values of the investigated parameters, depending on the level of exposure were noted, the earlier occurrence of the oxidative–antioxidative imbalance and changes in the values of markers of oxidative damage to these cellular macromolecules, as well as a lack of changes in some parameters at the low-level exposure and their occurrence at these same time points under the moderate treatment, show that the impact of this xenobiotic to some extent depended on the intensity of exposure and was stronger at the higher treatment. The finding that in the Cd_5_ group the value of OSI, which is the best marker of oxidative stress [44,59], was increased after the 3-month treatment, whereas at the lower exposure, this parameter was increased only after 10 months, indicated that the prooxidative action of cadmium depended on the level of exposure. Moreover, the negative and positive correlations between numerous evaluated variables and the concentration of cadmium in the brain, blood, and urine of the females that did not receive AE indicated the existence of a dependence between the prooxidative action of this heavy metal in the nervous tissue and the intensity of exposure to this xenobiotic and its accumulation in the brain. The lack of dose-related differences in the values of numerous parameters at the specific time points between the Cd_1_ group and the Cd_5_ group may be explained by no difference in the brain accumulation of cadmium in these animals despite higher median values of the concentration of this element in the females maintained on the 5 mg Cd/kg diet (Table S1) [39].

The prooxidative action of cadmium as a mechanism of its neurotoxicity has already been reported by some authors [15,16,17,18,19,20,21,22,23,24,25,26,27,28,29,30,31,32,33,34]; however, the current investigation is the first that showed that the treatment with this toxic element, reflecting the current low-level intoxication of inhabitants in developed countries, can be a cause of destroying the balance between the processes of oxidation and reduction in the nervous tissue. It is known that although cadmium is not able to create reactive oxygen species (ROS) and free radicals directly, it disturbs the oxidative–antioxidative balance by weakening the antioxidative barrier [5,17,21,31,34]. The research reported in this article disclosed that low-to-moderate exposure to this toxic element led to the oxidative–antioxidative imbalance in the nervous tissue via weakening its enzymatic and non-enzymatic antioxidative capacity and enhancing the amount of prooxidants such as MPO, XOD, and H_2_O_2_. The numerous positive and negative correlations between TAS, TOS, and OSI, which are the best indices of the balance between the processes of oxidation and reduction and other measured markers of the oxidative–antioxidative status in the females that received the Labofeed diet containing 0 (trace amounts of cadmium were present in the standard diet, but this element was not added to the diet), 1, and 5 mg Cd/kg and not supplemented with AE during this time, as well as the negative dependencies between the value of TAS and the TOS and OSI values and the positive correlation between TOS and OSI in these animals confirmed that the cadmium-induced attenuation of the antioxidative capacity and the increased concentrations of prooxidants contributed to destroying the oxidative–antioxidative status in the brain. It should be emphasized that regardless of the level of exposure to cadmium, the weakening of the antioxidative defence of the nervous tissue was preceded by the enhancement in its oxidative status.

Changes in the numerical values of various markers of the antioxidative and oxidative status of the nervous tissue, including a decrease in the activities of antioxidative enzymes (SOD, CAT, GPx, and GR) [17,21,28,29,33], a decline in the concentration of the main non-enzymatic antioxidants in the body, such as GSH and the GSH/GSSH ratio [15,16,17,18,31,34], as well as an increase in the concentration of ROS, including H_2_O_2_ [16,18,19,34], were reported by other authors. Moreover, oxidative damage to proteins (reflected in an elevated concentration of PC) [17] and an increase in the parameters indicating oxidative destruction of lipids (increased concentrations of thiobarbituric acid-reactive substances and MDA) [15,16,17,18,19,29,32] in the nervous tissue were noted. However, most of these effects were noted in animal models of high-level short-term exposure to this xenobiotic, which does not reflect the exposure currently occurring in humans [15,16,17,18,19,20,21,22,23,24,25,26,27,28,29,30,31,32,33]. The only available experimental study focused on cadmium neurotoxicity at low-level intoxication was carried out by Chouit et al. [21], who reported that exposure to 0.017 mg CdCl_2_/kg b.w. (0.01 mg Cd/kg b.w.) was a cause of destroying the balance between the processes of oxidation and reduction and enhancing apoptosis in the brain, as well as resulting in increased anxiety and decreased cognitive functions, but the exposure lasted only 60 days, and thus, unlike the present study, it did not reflect human environmental exposure lasting for the entire lifespan.

Regardless of the cause, the occurrence of oxidative stress in the brain results in the disruption of the proper functioning of the nervous system [15,16,17,23,26,29,34]. It has been reported that oxidative–antioxidative imbalance caused by cadmium, including a drop in the GSH concentration and GPx and SOD activities in the brain, as well as an increase in the generation of ROS and LPO, is connected with alterations in memory functioning [15,16,19]. Destroying the oxidative–antioxidative balance can also lead to the activation of destructive processes, including apoptotic pathways in the nervous system cells [15,16]. Moreover, an increase in the concentration of ROS due to intoxication with cadmium was reported to result in the destruction of mitochondria in the brain [60]. In a more recent study performed in the currently used experimental model, at both levels of exposure to cadmium, a reduction in the mitochondrial activity of MnSOD in the brain occurred after the shortest treatment and it remained throughout the whole experiment (the activity was decreased by 12–63%) [40]. Because the induction of oxidative stress is the possible mechanism by which cadmium mostly affects both the function and structure of neurons [16,17,18,23,25,26,28,29,30], the findings of the current study allowed for the conclusion that cadmium, even at low intoxication, may exert neurotoxic action via destroying the oxidative–antioxidative balance in the brain and oxidative modifications of lipids, proteins, and DNA; however, it needs further study. Moreover, the finding by Chouit et al. [21] of increased anxiety and decreased cognitive functions in rats due to lower and shorter exposure (0.01 mg Cd/kg b.w. for 60 days) than in the present study via the diet containing 1 mg Cd/kg (0.038–0.085 mg Cd/kg b.w.) allowed us to hypothesize that cadmium at the levels of treatment corresponding to the current exposure of the general population could also cause minor destroying effects in the functioning of the nervous system, which did not lead to any disturbances noticeable during daily observations of the animals. The fact that there were no signs of morbidity in the females treated with the 1 and 5 mg Cd/kg diet should not be surprising due to the low and moderate exposure to cadmium. However, the absence of any visible signs of toxicity did not allow us to exclude the presence of very subtle disorders that could not be detected based only on daily observations of the animal’s health.

A noteworthy result of this work is that at the moderate exposure to cadmium, an oxidative–reductive imbalance in the brain was noted after 3 months of the experiment, reflecting the first period of human life (7–14 years) in which the fastest development and growth occur and in which the nervous system is especially vulnerable to damage [27]. Moreover, a detailed analysis of the current findings together with the previous outcomes of our research group in these animals [44,45] revealed that the brain is more sensitive to the prooxidative activity of cadmium than the kidney and liver. After the treatment with the 5 mg Cd/kg diet for 3 months, the kidney OSI [45] was within the range of the control group; in the liver, the value of OSI was enhanced by 17% [44], whereas in the brain it was as much as 13-fold higher compared to the females from the control group. The value of OSI in the animals fed with the diet containing 1 mg Cd/kg remained unchanged in all of the above-mentioned tissues after 3 months [44,45], whereas, after 10 months, it was unaffected in the kidney [45] and higher by 43% in the liver [44] and 6.3-fold in the brain. The higher vulnerability of the brain to the prooxidative action of cadmium at low-to-moderate exposure, despite markedly lower retention of this trace element than in the main organs of its storage in the body (i.e., kidneys and liver) [39], may be explained by a large quantity of susceptible to oxidation polyunsaturated fatty acids, low antioxidative potential, and high usage of oxygen in this organ [5,10,38]. The measurements performed by our research team in the female rats also revealed that the antioxidative defence of the brain is weaker (lower values of TAS, GSH concentration, and the activities of CAT, GPx, and GR) compared to other organs like the kidneys and liver [44,45]. These outcomes, together with the already published and above-mentioned results of our research group in these same animals concerning the toxicity of cadmium and its accumulation in the body [39,44,45], which was the most effective during the first period of the animals’ life, suggest that this xenobiotic may be particularly dangerous to developing organisms in which the blood–brain barrier is not fully developed and thus is more permeable to xenobiotics.

Available literature data show that the state of oxidative–antioxidative imbalance in the brain and neurodegenerative disorders may be related to disturbances in the Trx system [61]. This system is the antioxidative system that regulates redox signalling in the brain, as well as performs anti-inflammatory and antiapoptotic actions [61]. It has been reported that disturbances in the Trx system in the brain can result in an elevation of the intensity of oxidative stress and that a deficiency of TrxR is connected with faster progression in Parkinson’s disease [61]. That is why when looking for the possible mechanisms of the neurotoxic action of cadmium we also considered the involvement of destruction of the Trx-dependent system in these pathways. The impact of cadmium on the Trx system (Trx, TrxR, and TPx) in the nervous tissue was assessed for the first time in the present study. Our results suggest that this xenobiotic at low-level exposure, unlike the moderate treatment, may exert a damaging impact on the nervous tissue via destroying the Trx system.

This study draws attention to the brain as an organ particularly sensitive to damage at repeated, even low, exposure to cadmium and the possible mechanisms of the neurotoxic action of this heavy metal. Notwithstanding, the main and, what is very important, having practical application outcome of this research is the finding, in the animal model of current environmental exposure to cadmium in developed nations, that the administration of a polyphenol-rich AE provided effective protection against this trace element-induced oxidative–antioxidative misbalance in the nervous tissue. The extract enhanced the antioxidative capacity of the brain and lowered the amount of ROS such as H_2_O_2_ and the levels of oxidases (XOD and MPO) that participate in ROS generation, and, as a result, reduced TOS and protected against destruction of the oxidative–antioxidative balance and the injury of proteins, lipids, and DNA in this organ. The fact that AE entirely protected against the majority of cadmium-caused modifications of the examined variables describing the redox status of the nervous tissue indicates that this extract may be effective in the alleviation of the toxic effect of cadmium on the nervous system via its antioxidative action.

The beneficial impact of AE on the nervous tissue under exposure to cadmium may be explained by its direct and indirect effects. The direct effect might result from the potent reductive capacity of numerous ingredients of the extract, as well as the capability of polyphenols to form stable complexes with divalent ions of cadmium (Cd^2+^) [7,43]. It is important to emphasize that we noted that the 0.1% aqueous AE is characterized by a high activity to scavenge free radicals [43]. Moreover, the preventive effect of AE on oxidative stress caused by cadmium in the kidneys, liver, bone, and salivary glands [42,43,44,45], revealed in the previous studies performed on these animals, confirmed its antioxidative properties. High antioxidative action is characteristic of polyphenolic compounds being the main components of AE, mainly anthocyanins consisting of 33% of all polyphenols present in the 0.1% extract with which the animals were supplemented [41,46,47,48,49,52]. However, the favourable effect of AE supplementation might also result from the presence of other ingredients, including pectins, triterpenes, carotenoids, vitamin C, and vitamin E, as well as minerals [47,48]. Because chokeberries and their products contain other ingredients known for antioxidative action in the brain, such as vitamin E, zinc, carotenoids, and selenium [33,62,63,64], the protective impact of the extract was probably not only the effect of polyphenols but also of the other ingredients characterized with reductive capacity. Moreover, it seems possible that because of their same mode of action, some components of the extract might act synergistically. Luís et al. [65] revealed that quercetin used together with chlorogenic acid or cyanidin-3-O-glucoside performed additive effects in scavenging free radicals. These compounds were present in the AE and thus their synergistic action cannot be excluded. What is more, compounds such as quercetin, vitamin E, and vitamin C, which all are present in chokeberries, not only are characterized by antioxidative capacity but also can form complexes with ions of trace metals and can facilitate them to act synergistically in the prevention of oxidative stress [66]. The indirect protective impact of the AE might also be related to the presence in the extract of tannins, which can complex ions of cadmium [67].

Substances that can reach the brain by penetrating the blood–brain barrier may perform direct action in this organ. The knowledge of the ability of polyphenols to cross the blood–brain barrier is very limited so far; however, it has been shown that one of the polyphenols present in the AE (chlorogenic acid) can cross this barrier [68]. Thus, the direct beneficial impact of the administration of AE might result, among others, from the action of this compound. Polyphenols that can penetrate the blood–brain barrier could exert beneficial effects not only due to their antioxidative properties but also by complexation of cadmium ions, which prevents their toxic action on the nervous tissue. It also seems possible that the favourable effect of AE on the balance between the processes of oxidation and reduction in the nervous tissue might be mediated by the influence of the extract ingredients on the Trx-dependent antioxidative system. Another polyphenolic ingredient of the extract that might contribute to its protective impact is quercetin, which was revealed to improve the functional status of the blood–brain barrier [69]. It seems that the role of quercetin in this protection may be important because cadmium damages the blood–brain barrier [5,27]. Moreover, vitamin E, as a lipid-soluble compound, can cross the blood–brain barrier [70] and thus it acts directly in the brain, whereas β-carotene not only can cross this barrier, but also protect against its damage [64].

Owing to our previous results of a study in the same animals [39,40], the protective outcome of supplementation with AE against the impact of cadmium on the nervous tissue might also, at least partially, especially for the long-term moderate exposure, result from the indirect action of the extract in reducing the accumulation of this xenobiotic in the brain. It has been reported that the administration of AE reduced cadmium absorption from the digestive tract, increased its urinary excretion, and thus decreased its retention in various organs, including the brain. In the females supplemented with AE under the long-term feeding with the 5 mg Cd/kg diet, the cadmium concentration in the nervous tissue of the brain origin was lower (by 22% and 20% after 17 and 24 months, respectively) [39] and in the brain mitochondria (by 23% after 24 months) [40] compared to females not supplemented with the extract during intoxication with this heavy metal. Although the application of chokeberry extract to the animals treated with the 1 mg Cd/kg diet had no impact on the concentration of cadmium in the brain as a whole organ [39], it prevented the retention of this element in brain mitochondria after 3 months of the study [40]. Since mitochondria are, on the one hand, the main source of ROS in cells, and on the other hand, are particularly vulnerable to injury by prooxidants [60], it cannot be excluded that the decrease in cadmium content in the mitochondria may be an important pathway in the mechanism of the indirect protection provided by AE against cadmium neurotoxicity. The positive correlations between the brain concentration of cadmium and the best redox biomarkers i.e., TOS and OSI, in the animals supplemented with AE indicates the existence of a dependence between this toxic element’s concentration in the nervous tissue and its prooxidative action, as well as shows that protection against this xenobiotic accumulation will improve the balance between the processes of oxidation and reduction in the nervous tissue.

It has been shown in experimental studies that some polyphenolic compounds present in the chokeberry extract, including quercetin [25] and kaempferol [16,29], as well as other polyphenol-rich products, such as extracts from strawberries [22], mango [20], grape seeds [23], and *F. Ferulla* [24], and the extract and juice from parsley [26,30], have protective action against cadmium-induced redox imbalance and its negative outcomes in the brain. However, our study provided the first evidence that AE may be effective in protecting against the damaging action of cadmium in the nervous tissue and that the adverse effects of the low-to-moderate treatment with this toxic heavy metal on the nervous tissue can be ameliorated or even prevented by co-administration of the extract.

Because the present study was conducted in an experimental in vivo model well reflecting the current lifespan exposure to cadmium in developed nations, these results may be extrapolated into humans and have important practical implications. Seeing that an adverse impact of cadmium was noted in female rats at exposure to the 1 mg Cd/kg diet, resulting in this trace metal concentration in the urine and blood within the levels currently determined in the world general population [5,14,51], these findings showed that greater consideration should be given to lifetime environmental cadmium exposure as a factor confounding the health of the general population, including a risk of nervous system damage. Based on these results, it can be assumed that the no observed adverse effect level (NOAEL) of exposure to cadmium for damage to the nervous system is lower than 39.2–83.8 µg/kg b.w. (such cadmium intake was recorded in the Cd_1_ group). Moreover, this study, together with our research team’s previous findings in this experimental model [39,40,41,42,43,44,45], provided strong evidence that AE is effective in protecting against various effects of cadmium toxicity. It creates the future possibility of using chokeberries and products based on these berries to protect against low-to-moderate environmental exposure to cadmium; however, this needs further studies.

### Limitations of the Study

Discussing the results of the present study, we want to emphasize that we are aware not only of its strengths but also of its limitations. One of them is not performing measurements in different parts of the brain such as the cerebrum, cerebellum, thalamus, hypothalamus, and brainstem. However, this was impossible due to the wide range of measurements planned to be carried out to evaluate the destroying influence of cadmium and the beneficial effect of AE on the nervous tissue. Thus, we decided to perform the measurements in nervous tissue of brain origin. The next limitation is conducting this research only on female animals, so the results can be interpreted only for the female organism. However, females are more vulnerable to the toxic action of cadmium [53] and thus female rats were more appropriate experimental animals to examine the effects of this xenobiotic under low-level exposure. Moreover, revealing the beneficial impact of AE in females treated with cadmium allowed us to hypothesize that this effect will also occur in males. Although our results undoubtedly indicated that even low-level intoxication with cadmium during the lifetime poses a risk of nervous system damage and the administration of chokeberry fruit extract may reduce this risk, currently, we cannot explain all of the mechanisms of the toxic impact of this xenobiotic or the protective effects of the aronia extract. We also cannot recognize which of the numerous biologically active ingredients present in chokeberries, apart from polyphenols, were responsible for the beneficial impact of the extract. However, this does not limit the importance and practical implications of our findings because we have investigated and revealed the effectiveness of the extract from *A. melanocarpa* berries as a whole. Moreover, we are very surprised by the finding of a decreased t-SH concentration in the nervous tissue after 3 months of the administration of AE alone; however, it was the only unfavourable and temporary effect noted in the case of supplementation with this extract without the treatment with cadmium. We are fully aware of the necessity of further research in this area and we are conducting such investigations, the results of which will be published soon. However, despite these limitations, our study allowed us not only to assess the toxic impact of cadmium alone on the redox status of the nervous tissue at low-to-moderate intoxication but also to propose an effective protective strategy. It seems very important for further studies focused on looking for a protective strategy against the impact on health of a common contaminant of diet such as cadmium that aronia products may be effective in this regard. They are nutritionally valuable and thus recommended as a functional food and are widely available on the market in various forms (juices, extracts, jams, etc.).

## 5. Conclusions

The results of the present study allowed for the conclusion that low-to-moderate-level repeated, and especially lifetime, intoxication with cadmium disturbs the balance between the processes of oxidation and reduction in the brain, resulting in the occurrence of oxidative stress and enhanced lipid peroxidation, as well as oxidative damage to proteins and DNA in the nervous tissue, and via these pathways may contribute to injury of the nervous system. Moreover, these findings show that regular intake of products based on *A. melanocarpa* fruit can protect against the oxidative–reductive imbalance caused by cadmium and its outcomes in the brain, and thus prevent nervous system impairment by this xenobiotic. In summary, even low-level chronic exposure to cadmium poses a risk of nervous system damage via prooxidative mechanisms, while the consumption of aronia-based nutritional products characterized by antioxidative properties seems to be an effective strategy for protecting the nervous tissue from the damaging action of this xenobiotic.

## Figures and Tables

**Table 1:** Relationships between total antioxidative status (TAS), total oxidative status (TOS), and oxidative stress index (OSI) and other indices of the oxidative–antioxidative status in the brain of female rats treated or not with 0.1% aqueous extract from *Aronia melanocarpa* L. berries (AE).

Parameter	Regression Analysis	TAS		TOS		OSI	
		Without AE	With AE	Without AE	With AE	Without AE	With AE
SOD	β*^p^* R^2^	NS	NS	−0.440 * 0.157	NS	NS	NS
CAT	β*^p^* R^2^	0.725 ^‡^ 0.502	NS	−0.780 ^‡^ 0.591	NS	−0.500 * 0.214	NS
GPx	β*^p^* R^2^	0.514 * 0.227	NS	−0.650 ^‡^ 0.399	NS	−0.470 * 0.187	NS
GR	β*^p^* R^2^	NS	0.577 ^†^ 0.302	0.493 * 0.205	NS	NS	NS
Trx	β*^p^* R^2^	NS	NS	NS	NS	NS	NS
TrxR	β*^p^* R^2^	NS	NS	NS	NS	NS	NS
TPx	β*^p^* R^2^	0.650 ^†^ 0.394	NS	−0.510 * 0.220	NS	NS	0.502 * 0.216
GSH	β*^p^* R^2^	0.471 * 0.182	NS	−0.410 ^#^ 0.129	NS	NS	NS
GSSG	β*^p^* R^2^	NS	NS	NS	NS	NS	NS
GSH/GSSG	β*^p^* R^2^	0.424 * 0.138	NS	NS	NS	NS	NS
t-SH	β*^p^* R^2^	0.742 ^‡^ 0.528	NS	−0.735 ^‡^ 0.517	NS	−0.505 * 0.218	NS
H_2_O_2_	β*^p^* R^2^	NS	NS	0.561 * 0.281	−0.500 * 0.213	NS	−0.520 ^†^ 0.237
XOD	β*^p^* R^2^	−0.480 * 0.193	NS	0.454 * 0.166	NS	NS	NS
MPO	β*^p^* R^2^	−0.740 ^‡^ 0.528	NS	0.687 ^‡^ 0.446	NS	0.488 * 0.200	NS
LPO	β*^p^* R^2^	−0.630 ^†^ 0.368	NS	0.743 ^‡^ 0.529	NS	0.676 ^‡^ 0.430	NS
8-iso	β*^p^* R^2^	−0.740 ^‡^ 0.524	NS	0.767 ^‡^ 0.568	NS	0.516 * 0.230	NS
3-NT	β*^p^* R^2^	−0.660 ^‡^ 0.401	NS	0.722 ^‡^ 0.497	NS	NS	NS
PC	β*^p^* R^2^	−0.450 * 0.163	0.473 * 0.187	0.765 ^‡^ 0.565	NS	NS	NS
ɣ-H2AX	β*^p^* R^2^	−0.590 ^†^ 0.320	NS	0.743 ^‡^ 0.530	NS	NS	−0.430 * 0.144

The results of the regression analysis are shown as the β coefficient and R^2^. The level of statistical significance (*p*) is marked as * *p* < 0.05, ^†^ *p* < 0.01, ^‡^ *p* < 0.001, and ^#^ *p* = 0.05, whereas NS means a lack of relationship. CAT, catalase; GPx, glutathione peroxidase; GR, glutathione reductase; GSH, reduced glutathione; GSSG, oxidized glutathione; GSH/GSSG, the ratio of reduced glutathione and oxidized glutathione; H_2_O_2_, hydrogen peroxide; MPO, myeloperoxidase; LPO, lipid peroxides; PC, protein carbonyl groups; SOD, superoxide dismutase; TPx, thioredoxin peroxidase; Trx, thioredoxin; TrxR, thioredoxin reductase; t-SH, total thiol groups; XOD, xanthine oxidase; ɣ-H2AX, ɣ-H2A histone family member X; 3-NT, 3-nitrotyrosine; 8-iso, 8-isoprostane.

**Table 2:** Relationships between markers of the oxidative–antioxidative status of the brain and cadmium (Cd) concentration in the brain, blood, and urine in rats treated or not with 0.1% aqueous extract from *Aronia melanocarpa* L. berries (AE).

Parameter	Regression Analysis	Cd in the Brain		Cd in the Blood		Cd in the Urine	
		Without AE	With AE	Without AE	With AE	Without AE	With AE
SOD	β*^p^* R^2^	−0.351 ^‡^ 0.114	NS	−0.268 ^†^ 0.062	NS	−0.283 ^†^ 0.070	NS
CAT	β*^p^* R^2^	−0.418 ^‡^ 0.166	NS	−0.423 ^‡^ 0.170	NS	−0.366 ^‡^ 0.125	NS
GPx	β*^p^* R^2^	−0.304 ^†^ 0.823	NS	−0.433 ^‡^ 0.179	NS	−0.351 ^‡^ 0.114	NS
GR	β*^p^* R^2^	NS	0.407 ^‡^ 0.156	NS	NS	NS	NS
Trx	β*^p^* R^2^	NS	NS	NS	NS	NS	NS
TrxR	β*^p^* R^2^	NS	NS	NS	−0.218 * 0.037	NS	NS
TPx	β*^p^* R^2^	NS	NS	NS	NS	NS	NS
GSH	β*^p^* R^2^	NS	0.346 ^‡^ 0.110	NS	0.389 ^‡^ 0.142	NS	0.399 ^‡^ 0.150
GSSG	β*^p^* R^2^	NS	NS	NS	0.257 * 0.056	NS	0.255 * 0.055
GSH/GSSG	β*^p^* R^2^	NS	NS	NS	NS	NS	NS
t-SH	β*^p^* R^2^	−0.273 ^†^ 0.064	NS	−0.325 ^†^ 0.096	0.205 * 0.031	−0.280 ^†^ 0.068	NS
TAS	β*^p^* R^2^	NS	NS	NS	NS	NS	NS
TOS	β*^p^* R^2^	0.363 ^‡^ 0.122	0.437 ^‡^ 0.182	0.513 ^‡^ 0.255	0.304 ^†^ 0.083	0.432 ^‡^ 0.178	0.387 ^‡^ 0.141
OSI	β*^p^* R^2^	0.378 ^‡^ 0.134	0.384 ^‡^ 0.138	0.495 ^‡^ 0.237	0.338 ^‡^ 0.105	0.432 ^‡^ 0.178	0.432 ^‡^ 0.178
H_2_O_2_	β*^p^* R^2^	NS	−0.413 ^‡^ 0.161	NS	−0.461 ^‡^ 0.204	NS	−0.462 ^‡^ 0.205
XOD	β*^p^* R^2^	0.233 * 0.044	NS	NS	NS	NS	NS
MPO	β*^p^* R^2^	0.299 ^†^ 0.795	NS	0.332 ^†^ 0.101	NS	0.353 ^‡^ 0.115	NS
LPO	β*^p^* R^2^	0.555 ^‡^ 0.301	0.221 * 0.039	0.530 * 0.273	0.251 * 0.052	0.464 ^‡^ 0.207	0.398 ^‡^ 0.150
8-iso	β*^p^* R^2^	NS	−0.328 ^†^ 0.098	0.316 ^†^ 0.090	NS	0.232 * 0.044	NS
3-NT	β*^p^* R^2^	0.213 * 0.035	NS	0.229 * 0.042	NS	0.229 * 0.042	NS
PC	β*^p^* R^2^	0.364 ^‡^ 0.123	NS	0.538 ^‡^ 0.282	NS	0.429 ^‡^ 0.175	NS
ɣ-H2AX	β*^p^* R^2^	0.272 ^†^ 0.064	NS	0.263 * 0.059	−0.229 * 0.042	NS	NS

The cadmium concentration in the brain, blood, and urine has been previously published [39]. The results of the regression analysis are shown as the β coefficient and R^2^. The level of statistical significance (*p*) is marked as * *p* < 0.05, ^†^ *p* < 0.01, and ^‡^ *p* < 0.001, whereas NS means a lack of relationship (*p* > 0.05). CAT, catalase; GPx, glutathione peroxidase; GR, glutathione reductase; GSH, reduced glutathione; GSSG, oxidized glutathione; GSH/GSSG, the ratio of reduced glutathione and oxidized glutathione; H_2_O_2_, hydrogen peroxide; MPO, myeloperoxidase; LPO, lipid peroxides; OSI, oxidative stress index (the ratio of TOS and TAS); PC, protein carbonyl groups; SOD, superoxide dismutase; TAS, total antioxidative status; TOS, total oxidative status; TPx, thioredoxin peroxidase; Trx, thioredoxin; TrxR, thioredoxin reductase; t-SH, total thiol groups; XOD, xanthine oxidase; ɣ-H2AX, ɣ-H2A histone family member X; 3-NT, 3-nitrotyrosine; 8-iso, 8-isoprostane.

## Data Availability

Data are contained within the article and Supplementary Materials. Further inquiries can be directed to the corresponding authors.

## Supplementary Materials

The following supporting information can be downloaded at: https://www.mdpi.com/article/10.3390/nu16040502/s1, Table S1: The concentration of cadmium (Cd) in the brain; Table S2: The absolute and relative weights of the brain of female rats; Table S3: The activities of superoxide dismutase (SOD) and catalase (CAT) in the brain of female rats; Table S4: The activities of glutathione peroxidase (GPx) and glutathione reductase (GR) in the brain of female rats; Table S5: The concentrations of thioredoxin (Trx), thioredoxin reductase (TrxR), and thioredoxin peroxidase (TPx) in the brain of female rats; Table S6: The concentrations of reduced glutathione (GSH) and oxidized glutathione (GSSG) and their ratio (GSH/GSSG) in the brain of female rats; Table S7: The concentration of the total thiol groups (t-SH) in the brain of female rats; Table S8: The total antioxidative status (TAS), total oxidative status (TOS), and the oxidative stress index (OSI) in the brain of female rats; Table S9: The concentrations of hydrogen peroxide (H_2_O_2_), myeloperoxidase (MPO), and xanthine oxidase (XOD) in the brain of female rats; Table S10: The concentrations of lipid peroxides (LPO) and 8-isoprostane (8-iso) in the brain of female rats; Table S11: The concentrations of 3-nitrotyrosine (3-NT), protein carbonyl groups (PC), and ɣ-H2A histone family member X (ɣ-H2AX) in the brain of female rats.

## Abbreviations

AE	*Aronia melanocarpa* L. berries extract
b.w.	body weight
CAT	catalase
Cd	cadmium
CV	coefficient of variation
DNA	deoxyribonucleic acid
ELISA	enzyme-linked immunosorbent assay
GPx	glutathione peroxidase
GR	glutathione reductase
GSH	reduced glutathione
GSSG	oxidized glutathione
GSH/GSSG	the ratio of reduced glutathione and oxidized glutathione
H_2_O_2_	hydrogen peroxide
MDA	malondialdehyde
MnSOD	manganese-dependent superoxide dismutase
MPO	myeloperoxidase
NADPH	nicotinamide adenine dinucleotide phosphate
LPO	lipid peroxides
OSI	oxidative stress index
PC	protein carbonyl groups
ROS	reactive oxygen species
SOD	superoxide dismutase
TAS	total antioxidative status
TOS	total oxidative status
TPx	thioredoxin peroxidase
Trx	thioredoxin
TrxR	thioredoxin reductase
t-SH	total thiol groups
XOD	xanthine oxidase
ɣ-H2AX	ɣ-H2A histone family member X
3-NT	3-nitrotyrosine
8-iso	8-isoprostane
