# Supplementary material for: The Protective Impact of Aronia melanocarpa L. Berries Extract against Prooxidative Cadmium Action in the Brain—A Study in an In Vivo Model of Current Environmental Human Exposure to This Harmful Element
Source: Nutrients. 2024 Feb 9;16(4):502. doi: 10.3390/nu16040502 (PMC10891719; doi:10.3390/nu16040502)
Supplement: Supplementary file 1 [file nutrients-16-00502-s001.zip › nutrients-2848778-supplementary.pdf]

nutrients

MDPI

Supplementary Materials

The Protective Impact of *Aronia melanocarpa* L. Berries Extract against Prooxidative Cadmium Action in the Brain—A Study in an In Vivo Model of Current Environmental Human Exposure to this Harmful Element

Agnieszka Ruczaj, Małgorzata M. Brzóska * and Joanna Rogalska

Department of Toxicology, Medical University of Bialystok, Adama Mickiewicza 2C Street, 15-222 Bialystok, Poland; agnieszka.ruczaj@sd.umb.edu.pl (A.R.); joanna.rogalska@umb.edu.pl (J.R.)
* Correspondence: malgorzata.brzoska@umb.edu.pl; Tel.: +48-85-748-5604; Fax: +48-85-748-5834

Table S1. The concentration of cadmium (Cd) in the brain.

Group	Experiment Duration			
	**3 Months**	**10 Months**	**17 Months**	**24 Months**
Control	0.026<br>0.014–0.041	0.028<br>0.022–0.039	0.017<br>0.012–0.026	0.025<br>0.014–0.038
AE	0.028<br>0.014–0.038	0.029<br>0.022–0.036	0.018<br>0.010–0.025	0.026<br>0.015–0.034
Cd_1	0.042 [a*]<br>0.027–0.057<br>↑ 62%	0.044 [a‡]<br>0.037–0.056<br>↑ 57%	0.031 [a*]<br>0.017–0.060<br>↑ 82%	0.048 [a‡]<br>0.026–0.071<br>↑ 92%
Cd_1 + AE	0.044 [a*]<br>0.021–0.062<br>↑ 69%	0.043 [a‡]<br>0.034–0.060<br>↑ 54%	0.030 [a*]<br>0.011–0.065<br>↑ 77%	0.048 [a‡]<br>0.027–0.067<br>↑ 92%
Cd_5	0.075 [a‡]<br>0.043–0.097<br>↑ 2.9x	0.051 [a‡]<br>0.039–0.065<br>↑ 82%	0.051 [a‡]<br>0.040–0.068<br>↑ 3x	0.056 [a‡]<br>0.050–0.065<br>↑ 2.3x
Cd_5 + AE	0.061 [a‡]<br>0.041–0.122<br>↑ 2.4x	0.043 [a‡]<br>0.035–0.056<br>↑ 54%	0.040 [a‡ e*]<br>0.036–0.046<br>↑ 2.4x ↘ 22%	0.045 [a‡ e*]<br>0.038–0.055<br>↑ 80% ↘ 20%

The table was prepared based on: Brzóska, M.M.; Gałażyn-Sidorczuk, M.; Jurczuk, M.; Tomczyk, M. Protective effect of *Aronia melanocarpa* polyphenols on cadmium accumulation in the body: A study in a rat model of human exposure to this metal. *Curr. Drug Targets* **2015**, *16*, 1470–1487. https://doi.org/10.2174/1389450116666150102121708. Rats were administered with 0, 1, or 5 mg Cd/kg of diet (groups: Control, Cd_1, and Cd_5) and/or 0.1% extract from *Aronia melanocarpa* L. berries (groups: AE, Cd_1 + AE, and Cd_5 + AE) for 3, 10, 17, and 24 months. Data are presented as a median and minimum and maximum value. The statistically significant differences compared to: a – Control group, and e – Cd_5 group are marked as * $p < 0.05$ and ‡ $p < 0.001$. ↑ – higher vs Control group, ↘ – lower vs Cd_5 group.

Table S2. The absolute and relative weights of the brain of female rats.

Group	Experiment Duration			
	3 Months	10 Months	17 Months	24 Months
**Absolute Weight of the Brain (g)**				
Control	1.721<br>1.380–1.943	1.894<br>1.798–2.038	2.017<br>1.844–2.250	2.055<br>1.846–2.096
AE	1.763<br>1.683–1.898	1.904<br>1.742–1.990	1.991<br>1.724–2.194	1.993<br>1.319–2.112
Cd_1	1.798<br>1.727–1.903	1.928<br>1.789–2.052	1.905<br>1.748–2.208	2.070<br>1.990–2.217
Cd_1 + AE	1.732<br>1.587–1.913	1.935<br>1.765–2.047	1.964<br>1.875–2.002	1.903<br>1.793–2.081
Cd_5	1.723<br>1.559–1.917	1.968<br>1.834–2.060	1.899<br>1.543–2.068	2.020<br>1.915–2.123
Cd_5 + AE	1.926<br>1.758–2.114	1.918<br>1.779–1.979	2.017<br>1.731–2.018	2.027<br>1.868–2.205
**Relative Weight of the Brain (g/100 g b.w.)**				
Control	0.552<br>0.399–0.604	0.433<br>0.421–0.509	0.411<br>0.301–0.517	0.313<br>0.267–0.381
AE	0.539<br>0.486–0.727	0.435<br>0.352–0.497	0.349<br>0.320–0.486	0.351<br>0.240–0.430
Cd_1	0.631<br>0.525–0.667	0.426<br>0.369–0.483	0.413<br>0.272–0.531	0.289<br>0.252–0.459
Cd_1 + AE	0.560<br>0.527–0.686	0.454<br>0.311–0.495	0.396<br>0.365–0.445	0.344<br>0.299–0.495
Cd_5	0.605<br>0.471–0.715	0.441<br>0.386–0.491	0.358<br>0.267–0.609	0.349<br>0.304–0.439
Cd_5 + AE	0.614<br>0.542–0.687	0.473<br>0.366–0.559	0.403<br>0.310–0.494	0.349<br>0.314–0.490

Rats were administered with 0, 1, or 5 mg of cadmium (Cd)/kg of diet (groups: Control, Cd_1, and Cd_5) and/or 0.1% extract from *Aronia melanocarpa* L. berries (AE) (groups: AE, Cd_1 + AE, and Cd_5 + AE) for 3, 10, 17, and 24 months. Data are presented as a median and minimum and maximum value for 8 rats in each group, except for 7 females in the AE, Cd_1, and Cd_5 groups after 24 months. No statistically significant differences (non-parametric Kruskal-Wallis test) between groups were noted.

Table S3. The activities of superoxide dismutase (SOD) and catalase (CAT) in the brain of female rats.

Group	Experiment Duration			
	**3 Months**	**10 Months**	**17 Months**	**24 Months**
	**SOD (U/mg protein)**			
Control	0.932<br>0.303–1.734	0.732<br>0.196–0.835	1.321<br>1.037–1.674	1.360<br>1.071–1.653
AE	0.913<br>0.651–2.118	0.743<br>0.570–1.222	1.355<br>1.086–2.366	1.393<br>0.954–2.445
Cd_1	0.505<br>0.247–0.909	0.633<br>0.168–0.849	1.284<br>0.994–2.101	1.326<br>0.805–2.027
Cd_1 + AE	0.872<br>0.554–1.312	0.869<br>0.567–1.104	1.178<br>0.985–1.815	1.270<br>1.094–2.067
Cd_5	0.721<br>0.293–1.190	0.608<br>0.158–0.934	0.880 $^{a* \, b\dagger \, c*}$<br>0.662–1.094	0.680 $^{a* \, b\dagger \, c* \, d*}$<br>0.644–0.928
Cd_5 + AE	1.078 ^{c*}<br>0.776–1.429	0.920 ^{e*}<br>0.689–1.164	1.195 ^{e*}<br>1.024–2.449	1.292 ^{e*}<br>1.056–2.060
Effect size (η^2)	0.193	0.176	0.298	0.330
	**CAT (mU/mg protein)**			
Control	17.71<br>13.77–38.571	13.59<br>8.54–21.85	15.17<br>8.97–24.50	16.34<br>11.91–20.50
AE	24.91<br>14.91–51.99	15.99<br>7.69–29.06	21.43<br>10.273–30.636	22.25<br>12.14–34.59
Cd_1	8.574 $^{a* \, b\ddagger}$<br>7.003–11.93	6.490 $^{a\# \, b\dagger}$<br>4.388–10.635	9.052 ^{b†}<br>5.342–14.76	7.330 $^{a\# \, b\dagger}$<br>3.623–9.596
Cd_1 + AE	21.05 ^{c†}<br>12.20–33.38	14.08 ^{c*}<br>8.602–18.53	17.51<br>9.265–30.41	19.07 ^{c*}<br>11.35–24.32
Cd_5	12.38 ^{b*}<br>9.92–18.75	6.893 ^{b*}<br>4.091–11.57	6.580 $^{a* \, b\ddagger \, d\dagger}$<br>4.377–8.028	6.020 $^{a* \, b\dagger \, d\dagger}$<br>4.012–7.136
Cd_5 + AE	25.66 $^{c\ddagger \, e*}$<br>16.62–27.18	18.75 $^{c\ddagger \, e\ddagger}$<br>10.73–23.68	16.58 ^{e*}<br>11.01–20.37	16.39 $^{c* \, e*}$<br>13.31–20.72
Effect size (η^2)	0.595	0.608	0622	0.624

Rats were administered with 0, 1, or 5 mg of cadmium (Cd)/kg of diet (groups: Control, Cd_1, and Cd_5) and/or 0.1% extract from *Aronia melanocarpa* L. berries (AE) (groups: AE, Cd_1 + AE, and Cd_5 + AE) for 3, 10, 17, and 24 months. Data are presented as a median and minimum and maximum value for 8 rats in each group, except for 7 females in the AE, Cd_1, and Cd_5 groups after 24 months. The statistically significant differences (non-parametric Kruskal-Wallis test) compared to: a – Control group, b – AE group, c – Cd_1 group, d – Cd_1 + AE group, and e – Cd_5 group are marked as * $p < 0.05$, † $p < 0.01$, ‡ $p < 0.001$, and $^\#$ $p = 0.07–0.08$. To evaluate the effect size for the differences between the experimental groups, eta squared (η^2) was calculated (for $\eta^2 \geq 0.14$ the effect size is large).

Table S4. The activities of glutathione peroxidase (GPx) and glutathione reductase (GR) in the brain of female rats.

Group	Experiment Duration			
	**3 Months**	**10 Months**	**17 Months**	**24 Months**
	**GPx (mU/mg protein)**			
Control	21.98<br>18.35–57.47	27.08<br>14.23–40.61	27.79<br>20.03–33.49	29.36<br>15.74–39.66
AE	22.08<br>14.05–30.43	43.41<br>19.00–46.75	26.50<br>20.92–32.59	29.71<br>21.46–35.14
Cd_1	15.34<br>10.47–20.12	13.55 ^{b*}<br>6.509–16.85	14.24<br>9.665–23.59	15.97<br>11.31–25.71
Cd_1 + AE	22.33<br>12.05–35.27	12.11<br>8.85–20.64	22.30<br>11.80–33.67	22.48<br>14.33–34.15
Cd_5	19.89<br>10.34–39.25	4.000 $^{a\ddagger\ b\dagger}$<br>2.310–5.650	13.91 $^{a*\ b*}$<br>8.122–18.00	10.78 $^{a\dagger\ b\dagger}$<br>7.190–13.69
Cd_5 + AE	30.22 ^{c†}<br>21.41–44.14	11.13 ^{b*}<br>10.58–20.28	31.74 $^{c\dagger\ e\ddagger}$<br>23.25–37.20	27.62 ^{e‡}<br>22.51–38.17
Effect size (η^2)	0.278	0.730	0.553	0.538
	**GR (mU/mg protein)**			
Control	8.791<br>4.144–14.121	6.530<br>4.923–12.16	8.659<br>7.140–16.01	7.018<br>3.469–7.871
AE	6.783<br>3.006–9.830	5.772<br>3.401–9.760	2.205 ^{a‡}<br>1.360–3.610	2.617 ^{a*}<br>1.114–3.131
Cd_1	7.729<br>3.860–10.28	4.225 ^{a†}<br>2.450–4.859	3.589 ^{a*}<br>2.190–6.910	7.078 ^{b*}<br>4.475–9.443
Cd_1 + AE	9.808<br>5.330–16.20	7.818 ^{c†}<br>4.542–9.462	3.732 ^{a*}<br>3.193–6.500	6.801 ^{b*}<br>5.702–10.31
Cd_5	5.170<br>4.680–11.76	5.779<br>4.283–8.231	8.205 $^{b\ddagger\ c*}$<br>6.850–9.030	9.943 $^{a*\ b\ddagger}$<br>8.296–14.15
Cd_5 + AE	6.534<br>4.320–9.280	5.851<br>4.616–10.71	3.614 $^{a*\ e*}$<br>3.100–7.830	5.386 ^{e†}<br>2.679–7.357
Effect size (η^2)	-	0.270	0.709	0.618

Rats were administered with a diet containing 0, 1, or 5 mg of cadmium (Cd)/kg (groups: Control, Cd_1, and Cd_5) and/or 0.1% extract from *Aronia melanocarpa* L. berries (AE) (groups: AE, Cd_1 + AE, and Cd_5 + AE) for 3, 10, 17, and 24 months. Data are presented as a median and minimum and maximum value for 8 rats in each group, except for 7 females in the AE, Cd_1, and Cd_5 groups after 24 months. The statistically significant differences (non-parametric Kruskal-Wallis test) compared to: a – Control group, b – AE group, c – Cd_1 group, and e – Cd_5 group are marked as * $p < 0.05$, † $p < 0.01$, and ‡ $p < 0.001$. To evaluate the effect size for the differences between the experimental groups, eta squared (η^2) was calculated (for $\eta^2 \geq 0.14$ the effect size is large).

Table S5. The concentrations of thioredoxin (Trx), thioredoxin reductase (TrxR), and thioredoxin peroxidase (TPx) in the brain of female rats.

Group	Experiment Duration			
	3 Months	10 Months	17 Months	24 Months
		**Trx (ng/mg protein)**		
Control	0.959<br>0.492–1.679	0.788<br>0.481–1.242	0.693<br>0.482–0.976	0.519<br>0.353–0.792
AE	0.868<br>0.680–2.282	0.807<br>0.565–1.297	0.715<br>0.541–0.944	0.503<br>0.305–0.798
Cd_1	0.762<br>0.545–0.790	0.661<br>0.564–0.947	$0.388^{a\dagger\ b*}$<br>0.298–0.563	$0.225^{a*\ b\dagger}$<br>0.152–0.315
Cd_1 + AE	0.755<br>0.645–2.389	0.667<br>0.473–1.004	0.459<br>0.392–0.766	0.381<br>0.131–0.680
Cd_5	0.951<br>0.717–1.350	0.724<br>0.533–1.006	0.516<br>0.093–0.871	0.455<br>0.116–0.669
Cd_5 + AE	1.027^{c*}<br>0.837–3.877	0.776<br>0.587–0.888	0.463<br>0.329–0.913	0.371<br>0.254–0.561
Effect size (η^2)	0.130	-	0.300	0.270
		**TrxR (ng/mg protein)**		
Control	1.283<br>0.771–2.641	1.147<br>0.692–3.955	1.699<br>0.944–2.314	1.582<br>0.983–3.528
AE	2.063<br>1.293–3.686	1.012<br>0.633–1.748	1.370<br>1.004–2.115	1.734<br>0.568–2.386
Cd_1	1.174<br>0.577–2.392	0.881<br>0.383–1.391	1.596<br>1.232–3.893	1.486<br>1.275–2.019
Cd_1 + AE	1.898<br>1.310–5.288	1.014<br>0.264–1.657	1.679<br>1.094–2.424	1.463<br>1.119–1.868
Cd_5	1.834<br>0.769–2.340	0.699<br>0.421–2.048	1.872<br>1.058–3.194	1.608<br>0.904–2.447
Cd_5 + AE	1.590<br>0.725–2.883	0.712<br>0.346–0.972	1.693<br>1.376–1.855	$0.599^{a*\ b*\ c*\ d*\ e\dagger}$<br>0.108–0.820
Effect size (η^2)	-	-	-	0.330
		**TPx (ng/mg protein)**		
Control	2.774<br>1.832–19.28	2.579<br>1.500–6.679	3.360<br>2.193–3.687	3.232<br>2.415–4.988
AE	2.567<br>1.433–8.158	$1.026^{a\dagger}$<br>0.691–1.568	1.793<br>0.405–7.117	2.951<br>1.971–4.112
Cd_1	1.166<br>0.628–4.344	1.326<br>0.922–4.835	$1.093^{a\dagger}$<br>0.338–1.772	1.389^{a*}<br>1.120–1.982
Cd_1 + AE	4.821<br>1.373–7.572	1.737<br>0.923–4.132	$3.098^{c\dagger}$<br>2.363–4.840	$3.649^{c\dagger}$<br>2.572–4.748
Cd_5	2.597<br>1.444–4.972	1.959<br>0.888–3.264	2.256<br>1.616–2.667	2.294<br>1.566–2.751
Cd_5 + AE	2.703<br>1.879–6.734	1.560<br>1.319–2.372	2.857^{c*}<br>1.942–4.871	$4.501^{c\ddagger\ e\dagger}$<br>3.205–6.618
Effect size (η^2)	-	0.290	0.380	0.640

Rats were administered with a diet containing 0, 1, or 5 mg of cadmium (Cd)/kg (groups: Control, Cd_1, and Cd_5) and/or 0.1% extract from *Aronia melanocarpa* L. berries (AE) (groups: AE, Cd_1 + AE, and Cd_5 + AE) for 3, 10, 17, and 24 months. Data are presented as a median and minimum and maximum value for 8 rats in each group, except for 7 females in the AE, Cd_1, and Cd_5 groups after 24 months. The statistically significant differences (non-parametric Kruskal-Wallis test) compared to: a – Control group, b – AE group, c – Cd_1 group, d – Cd_1 + AE group, and e – Cd_5 group are marked as * $p < 0.05$, † $p < 0.01$, and ‡ $p < 0.001$. To evaluate the effect size for the differences between the experimental groups, eta squared (η^2) was calculated (for $\eta^2 \geq 0.14$ the effect size is large, for $0.01 < \eta^2 < 0.14$ it is medium).

Table S6. The concentrations of reduced glutathione (GSH) and oxidized glutathione (GSSG) and their ratio (GSH/GSSG) in the brain of female rats.

**Group**	**Experiment Duration**			
	**3 Months**	**10 months**	**17 Months**	**24 Months**
		**GSH (nmol/mg protein)**		
Control	4.090<br>2.741–5.520	2.247<br>1.530–3.381	1.472<br>1.323–2.195	0.869<br>0.845–1.252
AE	2.159<br>1.276–4.809	2.428<br>1.778–3.198	1.358<br>0.913–1.642	2.178 ^{a*}<br>1.596–4.228
Cd_1	1.046 ^{a†}<br>0.464–2.214	2.088<br>1.120–3.301	1.323<br>0.735–1.905	0.545 $^{b\dagger\ c\dagger}$<br>0.475–0.691
Cd_1 + AE	2.370<br>1.038–4.586	2.079<br>1.518–3.155	1.502<br>1.195–2.635	2.010 ^{c†}<br>1.424–4.172
Cd_5	2.032<br>1.323–3.871	1.288<br>0.498–4.810	1.454<br>0.767–1.592	0.499 $^{b\dagger\ d\dagger}$<br>0.325–1.131
Cd_5 + AE	4.000 ^{c‡}<br>3.339–6.165	4.438 $^{a*\ c\dagger\ d\dagger\ e\ddagger}$<br>3.496–5.806	2.525 $^{a*\ b\dagger\ c\dagger\ e*}$<br>2.200–3.251	1.866 $^{c\dagger\ e\dagger}$<br>1.165–3.704
Effect size (η^2)	0.505	0.370	0.361	0.773
		**GSSG (nmol/mg protein)**		
Control	2.796<br>2.402–5.520	1.596<br>1.054–2.306	0.692<br>0.560–1.033	0.722<br>0.570–1.993
AE	1.010 ^{a*}<br>0.568–1.899	1.178<br>1.042–1.706	0.628<br>0.393–1.075	0.380<br>0.243–0.550
Cd_1	1.046 ^{a†}<br>0.522–1.388	1.385<br>1.063–1.741	0.791<br>0.330–1.230	2.215 ^{b†}<br>0.825–2.553
Cd_1 + AE	0.486 ^{a‡}<br>0.201–2.118	0.780 ^{a*}<br>0.452–1.521	0.666<br>0.549–1.122	0.311 ^{c‡}<br>0.177–0.751
Cd_5	1.410 ^{a*}<br>0.605–2.390	1.550 ^{d*}<br>1.042–2.546	1.166 ^{a*}<br>0.857–1.540	0.721<br>0.451–2.162
Cd_5 + AE	1.613<br>0.946–2.036	1.831 ^{d‡}<br>1.367–2.439	1.053 $^{b\dagger\ d*}$<br>0.337–2.633	0.229 $^{a\dagger\ c\ddagger\ e*}$<br>0.126–0.475
Effect size (η^2)	0.503	0.392	0.297	0.717
		**GSH/GSSG**		
Control	1.000<br>1.000–2.122	1.311<br>1.000–2.602	2.188<br>1.839–2.679	1.204<br>0.430–2.089
AE	2.138<br>2.073–3.451	2.093<br>1.042–2.133	2.108<br>1.527–2.989	7.345<br>2.901–12.88
Cd_1	1.451<br>0.396–2.192	1.567<br>1.027–2.117	2.144<br>0.843–2.229	0.297 ^{b†}<br>0.202–0.635
Cd_1 + AE	2.527 ^{a*}<br>1.417–7.472	2.486<br>2.046–5.982	2.137<br>2.018–3.711	7.314 ^{c†}<br>4.579–9.628
Cd_5	2.267<br>0.838–6.584	0.971 ^{d*}<br>0.334–3.496	1.214 $^{a*\ d*}$<br>0.632–1.765	0.722 $^{b*\ d\dagger}$<br>0.231–1.415
Cd_5 + AE	2.259<br>2.109–6.383	2.565 ^{e*}<br>1.874–2.944	2.333 ^{e†}<br>1.235–7.626	8.300 $^{a*\ c\ddagger\ e\dagger}$<br>6.052–11.53
Effect size (η^2)	0.300	0.264	0.322	0.789

Rats were administered with a diet containing 0, 1, or 5 mg of cadmium (Cd)/kg (groups: Control, Cd_1, and Cd_5) and/or 0.1% extract from *Aronia melanocarpa* L. berries (AE) (groups: AE, Cd_1 + AE, and Cd_5 + AE) for 3, 10, 17, and 24 months. Data are presented as a median and minimum and maximum value for 8 rats in each group, except for 7 females in the AE, Cd_1, and Cd_5 groups after 24 months. The statistically significant differences (non-parametric Kruskal-Wallis test) compared to: a – Control group, b – AE group, c – Cd_1 group, d – Cd_1 + AE group, and e – Cd_5 group are marked as * $p < 0.05$, † $p < 0.01$, and ‡ $p < 0.001$. To evaluate the effect size for the differences between the experimental groups, eta squared (η^2) was calculated (for $\eta^2 \geq 0.14$ the effect size is large).

Table S7. The concentration of total thiol groups (t-SH) in the brain of female rats.

Group	Experiment Duration			
	**3 Months**	**10 Months**	**17 Months**	**24 Months**
	**t-SH (nmol/mg protein)**			
Control	89.44<br>53.41–154.1	63.31<br>39.39–74.43	51.33<br>43.51–64.62	62.53<br>45.21–92.53
AE	21.27 ^{a†}<br>10.58–125.6	32.39<br>20.78–77.09	43.22<br>34.34–75.51	28.07<br>14.68–145.3
Cd_1	32.60 ^{a†}<br>19.79–40.86	22.54 ^{a†}<br>15.42–47.54	27.73 $^{a† b*}$<br>18.65–34.02	20.15 ^{a*}<br>10.28–25.97
Cd_1 + AE	62.10<br>37.84–113.9	51.42 ^{c†}<br>40.08–84.94	44.48 ^{c*}<br>35.31–79.39	81.49 ^{c†}<br>50.41–107.3
Cd_5	64.11<br>21.47–116.7	27.18 $^{a† d*}$<br>17.66–51.13	25.32 $^{a† b* d*}$<br>19.17–34.52	21.52 $^{a* d†}$<br>13.54–25.09
Cd_5 + AE	56.36<br>42.89–71.34	42.30<br>36.10–69.51	52.66 $^{c† e†}$<br>43.97–75.79	88.71 $^{c† e†}$<br>42.01–116.9
Effect size (η^2)	0.430	0.490	0.650	0.620

Rats were administered with a diet containing 0, 1, or 5 mg of cadmium (Cd)/kg (groups: Control, Cd_1, and Cd_5) and/or 0.1% extract from *Aronia melanocarpa* L. berries (AE) (groups: AE, Cd_1 + AE, and Cd_5 + AE) for 3, 10, 17, and 24 months. Data are presented as a median and minimum and maximum value for 8 rats in each group, except for 7 females in the AE, Cd_1, and Cd_5 groups after 24 months. The statistically significant differences (non-parametric Kruskal-Wallis test) compared to: a – Control group, b – AE group, c – Cd_1 group, d – Cd_1 + AE group, and e – Cd_5 group are marked as * $p < 0.05$, and † $p < 0.01$. To evaluate the effect size for the differences between the experimental groups, eta squared (η^2) was calculated (for $\eta^2 \geq 0.14$ the effect size is large).

Table S8. The total antioxidative status (TAS), total oxidative status (TOS), and the oxidative stress index (OSI) in the brain of female rats.

Group	Experiment Duration			
	3 Months	10 Months	17 Months	24 Months
		**TAS (μmol/g protein)**		
Control	135.3<br>121.1–247.6	96.70<br>82.04–149.6	61.18<br>50.49–98.18	55.81<br>40.89–74.97
AE	136.7<br>106.0–340.5	100.0<br>80.70–137.3	85.32<br>61.59–107.2	52.55<br>44.28–97.41
Cd_1	108.4 ^{a*}<br>84.26–128.1	98.53<br>88.96–124.0	54.10<br>46.05–73.98	31.01 ^{a*}<br>15.96–34.45
Cd_1 + AE	109.3<br>100.7–311.3	94.53<br>80.44–143.6	69.80<br>58.10–104.9	88.65 ^{c‡}<br>80.92–110.3
Cd_5	133.0<br>105.3–183.7	94.71<br>80.12–130.2	44.60 $^{a*\,b\ddagger\,d*}$<br>28.97–47.68	28.50 $^{a\dagger\,d\ddagger}$<br>8.996–45.807
Cd_5 + AE	130.4<br>127.0–173.7	105.4<br>57.85–131.6	87.10 ^{e‡}<br>54.75–95.16	46.23 ^{e*}<br>33.90–95.49
Effect size (η^2)	0.198	-	0.564	0.697
		**TOS (μmol/g protein)**		
Control	3.780<br>2.138–4.934	2.535<br>1.892–3.530	2.268<br>1.734–4.439	1.963<br>1.284–3.595
AE	2.637<br>1.985–4.207	2.222<br>1.873–2.670	2.363<br>1.427–3.099	1.697<br>0.577–1.871
Cd_1	7.308<br>5.357–9.292	18.14 $^{a\dagger\,b\dagger}$<br>3.272–53.94	13.85 $^{a\dagger\,b\dagger}$<br>8.168–26.10	9.893 $^{a*\,b\dagger}$<br>7.583–14.16
Cd_1 + AE	6.044<br>4.552–10.52	3.875<br>1.137–16.64	3.622 ^{c†}<br>2.174–6.139	2.470 ^{c*}<br>1.295–3.450
Cd_5	37.11 $^{a\ddagger\,b\ddagger}$<br>10.76–89.60	17.69 $^{a\dagger\,b\ddagger}$<br>6.555–41.34	17.38 $^{a\ddagger\,b\ddagger}$<br>10.65–30.77	14.79 $^{a\dagger\,b\ddagger\,d*}$<br>10.43–23.61
Cd_5 + AE	12.65 $^{a*\,b\dagger\,e*}$<br>1.660–18.10	9.447 ^{b*}<br>5.015–16.62	3.053 $^{c*\,e\dagger}$<br>1.461–4.791	1.897 ^{e†}<br>0.968–3.683
Effect size (η^2)	0.747	0.626	0.709	0.688
		**OSI**		
Control	0.021<br>0.017–0.037	0.024<br>0.019–0.042	0.034<br>0.019–0.076	0.038<br>0.018–0.071
AE	0.022<br>0.010–0.025	0.023<br>0.014–0.028	0.025<br>0.015–0.037	0.032<br>0.010–0.038
Cd_1	0.071<br>0.048–0.073	0.151 $^{a*\,b\dagger}$<br>0.037–0.606	0.252 $^{a*\,b\ddagger}$<br>0.131–0.465	0.319 $^{a*\,b\dagger}$<br>0.248–0.887
Cd_1 + AE	0.051<br>0.020–0.097	0.041 ^{c*}<br>0.014–0.178	0.050 ^{c†}<br>0.026–0.100	0.025 ^{c†}<br>0.015–0.040
Cd_5	0.281 $^{a\ddagger\,b\ddagger}$<br>0.062–0.659	0.159 $^{a\ddagger\,b\ddagger}$<br>0.117–0.331	0.418 $^{a\dagger\,b\ddagger\,d\dagger}$<br>0.252–0.697	0.643 $^{a\dagger\,b\ddagger\,d\dagger}$<br>0.229–2.368
Cd_5 + AE	0.098 $^{a*\,b*}$<br>0.010–0.138	0.098 $^{b*\,e*}$<br>0.048–0.156	0.038 ^{e†}<br>0.015–0.056	0.037 ^{e†}<br>0.029–0.056
Effect size (η^2)	0.660	0.656	0.735	0.684

Rats were administered with a diet containing 0, 1, or 5 mg of cadmium (Cd)/ kg (groups: Control, Cd_1, and Cd_5) and/or 0.1% extract from *Aronia melanocarpa* L. berries (AE) (groups: AE, Cd_1 + AE, and Cd_5 + AE) for 3, 10, 17, and 24 months. Data are presented as a median and minimum and maximum value for 8 rats in each group, except for 7 females in the AE, Cd_1, and Cd_5 groups after 24 months. The statistically significant differences (non-parametric Kruskal-Wallis test) compared to: a – Control group, b – AE group, c – Cd_1 group, d – Cd_1 + AE group, and e – Cd_5 group are marked as * $p < 0.05$, † $p < 0.01$, and ‡ $p < 0.001$. To evaluate the effect size for the differences between the experimental groups, eta squared (η^2) was calculated (for $\eta^2 \geq 0.14$ the effect size is large).

Table S9. The concentrations of hydrogen peroxide (H_2O_2), myeloperoxidase (MPO), and xanthine oxidase (XOD) in the brain of female rats.

**Group**	**Experiment Duration**			
	**3 Months**	**10 Months**	**17 Months**	**24 Months**
**H_2O_2 (nmol/mg protein)**				
Control	4.251<br>1.297–8.331	3.684<br>2.047–4.870	3.374<br>2.814–5.460	3.830<br>2.754–4.309
AE	4.436<br>3.072–4.852	3.206<br>1.737–3.554	3.460<br>2.747–4.006	2.848<br>2.428–3.331
Cd$_1$	4.283<br>2.989–7.202	3.501<br>3.247–4.856	3.504<br>1.552–5.736	4.064 ^{b*}<br>3.584–4.581
Cd$_1$ + AE	1.748 $^{a*\ b\dagger\ c\dagger}$<br>0.652–2.823	2.357 $^{a*\ c\dagger}$<br>0.573–3.697	2.848<br>1.610–3.976	2.244 $^{a\dagger\ c\dagger}$<br>1.616–3.588
Cd$_5$	3.634<br>2.095–5.555	2.195 ^{c*}<br>1.559–4.103	4.640 $^{a*\ b\dagger\ c*\ d\dagger}$<br>3.887–5.084	4.629 $^{a*\ d\dagger}$<br>3.418–5.189
Cd$_5$ + AE	2.065<br>0.941–4.555	1.717 $^{a\ddagger\ b\ddagger\ c\ddagger}$<br>0.158–2.957	2.045 $^{a\ddagger\ b\ddagger\ c\dagger\ e\ddagger}$<br>1.496–2.212	1.849 $^{a\ddagger\ c*\ e\ddagger}$<br>1.059–2.502
Effect size (η^2)	0.410	0.380	0.490	0.750
**MPO (ng/mg protein)**				
Control	1.369<br>0.679–2.192	1.117<br>0.728–1.411	0.961<br>0.780–1.182	1.018<br>0.692–1.429
AE	1.215<br>1.089–2.260	1.091<br>0.447–1.520	0.928<br>0.732–1.261	1.171<br>0.719–1.282
Cd$_1$	1.171<br>1.008–1.401	1.507<br>1.005–2.756	1.250<br>0.717–1.380	1.918 ^{a†}<br>1.659–2.549
Cd$_1$ + AE	1.149<br>0.628–3.700	0.974<br>0.500–1.469	0.746<br>0.598–1.160	0.932 ^{c†}<br>0.561–1.288
Cd$_5$	1.364<br>0.918–1.908	1.795 $^{a\#\ d*}$<br>1.192–4.796	1.431 ^{d†}<br>1.060–1.909	1.824 $^{a*\ d\dagger}$<br>1.684–2.367
Cd$_5$ + AE	1.284<br>0.851–1.675	1.336<br>0.740–2.271	0.871 ^{e†}<br>0.539–0.998	1.064 $^{c*\ e*}$<br>0.879–1.246
Effect size (η^2)	-	0.300	0.410	0.600
**XOD (ng/mg protein)**				
Control	1.459<br>0.717–2.526	0.683<br>0.589–1.006	0.535<br>0.183–1.050	0.470<br>0.197–0.639
AE	1.209<br>0.688–2.565	0.640<br>0.553–0.965	0.598<br>0.141–0.736	0.502<br>0.261–1.584
Cd$_1$	1.495<br>0.901–2.162	0.722<br>0.643–1.427	0.665<br>0.394–2.621	0.841 $^{a\#}$<br>0.714–1.997
Cd$_1$ + AE	1.100<br>0.946–2.613	0.793<br>0.600–1.036	0.447<br>0.141–1.147	0.288 ^{c†}<br>0.123–0.533
Cd$_5$	1.307<br>0.964–2.129	0.803<br>0.708–1.062	0.965 ^{d*}<br>0.622–2.684	0.950 $^{a\#\ d\dagger}$<br>0.672–1.182
Cd$_5$ + AE	1.062<br>0.568–1.208	0.794<br>0.349–0.923	0.289 ^{e‡}<br>0.136–0.869	0.411 $^{c\dagger\ e*}$<br>0.146–0.538
Effect size (η^2)	-	-	0.340	0.580

Rats were administered with a diet containing 0, 1, or 5 mg of cadmium (Cd)/ kg (groups: Control, Cd$_1$, and Cd$_5$) and/or 0.1% extract from *Aronia melanocarpa* L. berries (AE) (groups: AE, Cd$_1$ + AE, and Cd$_5$ + AE) for 3, 10, 17, and 24 months. Data are presented as a median and minimum and maximum value for 8 rats in each group, except for 7 females in the AE, Cd$_1$, and Cd$_5$ groups after 24 months. The statistically significant differences (non-parametric Kruskal-Wallis test) compared to: a – Control group, b – AE group, c – Cd$_1$ group, d – Cd$_1$ + AE group, and e – Cd$_5$ group are marked as * $p < 0.05$, † $p < 0.01$, ‡ $p < 0.001$, and $^{\#}$ $p = 0.05–0.08$. To evaluate the effect size for the differences between the experimental groups, eta squared (η^2) was calculated (for $\eta^2 \geq 0.14$ the effect size is large).

Table S10. The concentrations of lipid peroxides (LPO) and 8-isoprostane (8-iso) in the brain of female rats.

Group	Experiment Duration			
	**3 Months**	**10 Months**	**17 Months**	**24 Months**
	**LPO (µmol/mg protein)**			
Control	0.787<br>0.490–0.925	0.598<br>0.351–1.103	0.678<br>0.532–0.709	0.573<br>0.487–0.646
AE	0.609<br>0.286–0.795	0.733<br>0.487–1.051	0.632<br>0.410–1.138	0.223 ^{a*}<br>0.165–0.275
Cd$_1$	1.256 ^{b*}<br>0.686–1.812	1.181<br>0.534–1.722	0.940<br>0.663–1.619	0.808 ^{b‡}<br>0.719–1.211
Cd$_1$ + AE	0.684<br>0.448–1.472	0.465 ^{c*}<br>0.203–1.204	0.581 ^{c*}<br>0.469–0.796	0.320 $^{c\#}$<br>0.209–0.449
Cd$_5$	1.589 $^{b\dagger\ d*}$<br>0.716–2.353	1.015 ^{d*}<br>0.521–1.926	0.984 $^{a*\ d\dagger}$<br>0.876–1.348	1.502 $^{b\ddagger\ d\dagger}$<br>1.332–2.172
Cd$_5$ + AE	1.420 ^{b†}<br>0.741–2.514	0.983<br>0.451–1.272	0.662 ^{e*}<br>0.327–0.915	0.307 $^{c*\ e\ddagger}$<br>0.190–0.559
Effect size (η^2)	0.510	0.240	0.390	0.820
	**8-iso (pg/mg protein)**			
Control	12.99<br>10.63–19.79	15.32<br>12.63–20.88	17.66<br>14.83–20.57	9.943<br>9.483–11.81
AE	12.02<br>7.715–17.20	14.08<br>10.95–25.70	18.91<br>9.557–21.06	18.94<br>11.50–21.70
Cd$_1$	11.97<br>9.776–14.63	18.21<br>15.60–24.08	28.30 $^{a\dagger\ b*}$<br>27.01–46.02	28.49 ^{a*}<br>23.84–31.81
Cd$_1$ + AE	11.02<br>10.61–15.74	11.83 ^{c*}<br>9.303–24.25	16.04 ^{c‡}<br>11.85–17.05	9.630 ^{c†}<br>7.492–11.59
Cd$_5$	15.73<br>12.77–20.70	19.11<br>13.58–24.91	26.02 ^{d†}<br>21.00–29.09	30.18 $^{a\dagger\ d\dagger}$<br>25.61–36.40
Cd$_5$ + AE	11.38<br>10.50–18.71	14.89<br>9.614–17.85	17.87 $^{c\ddagger\ e*}$<br>8.483–19.77	9.694 $^{c\dagger\ e\ddagger}$<br>8.344–11.72
Effect size (η^2)	-	0.210	0.700	0.770

Rats were administered with a diet containing 0, 1, or 5 mg of cadmium (Cd)/kg (groups: Control, Cd$_1$, and Cd$_5$) and/or 0.1% extract from *Aronia melanocarpa* L. berries (AE) (groups: AE, Cd$_1$ + AE, and Cd$_5$ + AE) for 3, 10, 17, and 24 months. Data are presented as a median and minimum and maximum value for 8 rats in each group, except for 7 females in the AE, Cd$_1$, and Cd$_5$ groups after 24 months. The statistically significant differences (non-parametric Kruskal-Wallis test) compared to: a – Control group, b – AE group, c – Cd$_1$ group, d – Cd$_1$ + AE group, and e – Cd$_5$ group are marked as * $p < 0.05$, † $p < 0.01$, ‡ $p < 0.001$, and $^\#$ $p = 0.05$. To evaluate the effect size for the differences between the experimental groups, eta squared (η^2) was calculated (for $\eta^2 \geq 0.14$ the effect size is large).

Table S11. The concentrations of 3-nitrotyrosine (3-NT), protein carbonyl groups (PC), and ɣ-H2A histone family member X (ɣ-H2AX) in the brain of female rats.

**Group**	**Experiment Duration**			
	**3 Months**	**10 Months**	**17 Months**	**24 Months**
**3-NT (ng/mg protein)**				
Control	101.1<br>17.05–122.5	71.05<br>56.72–125.5	75.76<br>23.59–87.31	47.61<br>33.15–61.64
AE	96.34<br>26.64–294.4	72.77<br>54.04–95.23	77.25<br>75.49–97.30	49.29<br>32.85–77.49
Cd_1	92.67<br>81.16–112.0	78.00<br>56.80–100.9	89.75<br>60.65–178.5	100.9 ^{a*}<br>61.26–107.4
Cd_1 + AE	69.00<br>28.03–237.5	71.89<br>45.98–102.4	65.28<br>34.93–74.01	47.68 ^{c*}<br>28.84–63.81
Cd_5	94.39<br>41.80–169.1	87.42<br>63.67–115.2	88.63 ^{d*}<br>66.64–109.3	93.35 $^{a*\ d*}$<br>65.47–114.1
Cd_5 + AE	86.51<br>55.24–147.6	68.64<br>58.98–102.5	52.05 $^{b*\ c\dagger\ e\dagger}$<br>36.68–63.20	47.44 $^{c*\ e*}$<br>33.38–66.44
Effect size (η^2)	-	-	0.420	0.520
**PC (nmol/mg protein)**				
Control	10.44<br>8.332–14.90	6.659<br>4.400–7.130	4.845<br>4.047–5.739	11.53<br>10.16–16.19
AE	9.393<br>6.815–16.53	7.808<br>5.798–8.759	5.097<br>4.295–8.959	10.83<br>9.722–14.42
Cd_1	9.407<br>7.393–21.10	6.372<br>5.459–10.76	10.88 ^{a*}<br>5.316–16.94	21.76 ^{b*}<br>16.48–29.70
Cd_1 + AE	8.495<br>6.546–11.82	5.144<br>3.842–8.789	4.677<br>3.908–7.967	17.96<br>10.53–29.38
Cd_5	13.34<br>8.564–17.15	10.97 $^{a*\ c*\ d\ddagger}$<br>7.030–14.09	23.44 $^{a\ddagger\ b\dagger\ d\ddagger}$<br>17.72–30.20	48.71 $^{a\ddagger\ b\ddagger}$<br>29.60–52.89
Cd_5 + AE	8.455<br>6.406–10.12	7.663<br>5.998–8.638	5.905 ^{e*}<br>5.202–7.623	14.96 ^{e†}<br>8.750–16.89
Effect size (η^2)	-	0.390	0.640	0.650
**ɣ-H2AX (ng/mg protein)**				
Control	1.007<br>0.371–1.425	0.873<br>0.638–1.009	0.992<br>0.752–1.369	0.777<br>0.489–0.986
AE	1.194<br>0.368–3.263	0.840<br>0.697–1.226	0.920<br>0.658–1.084	0.919<br>0.251–1.087
Cd_1	0.836<br>0.326–1.160	0.835<br>0.673–1.282	1.016<br>0.888–1.819	1.534 $^{a\#}$<br>1.191–2.111
Cd_1 + AE	0.979<br>0.888–1.362	0.851<br>0.637–1.276	0.817<br>0.697–1.343	0.635 ^{c†}<br>0.249–1.002
Cd_5	1.141<br>0.457–1.341	0.783<br>0.647–1.120	1.147<br>0.781–1.813	1.622 $^{a*\ d\dagger}$<br>1.299–1.915
Cd_5 + AE	0.944<br>0.480–1.122	0.867<br>0.726–0.946	0.777<br>0.276–1.009	0.616 $^{c\dagger\ e\ddagger}$<br>0.483–0.694
Effect size (η^2)	-	-	-	0.640

Rats were administered with a diet containing 0, 1, or 5 mg of cadmium (Cd)/kg (groups: Control, Cd_1, and Cd_5) and/or 0.1% extract from *Aronia melanocarpa* L. berries (AE) (groups: AE, Cd_1 + AE, and Cd_5 + AE) for 3, 10, 17, and 24 months. Data are presented as a median and minimum and maximum value for 8 rats in each group, except for 7 females in the AE, Cd_1, and Cd_5 groups after 24 months. The statistically significant differences (non-parametric Kruskal-Wallis test) compared to: a – Control group, b – AE group, c – Cd_1 group, d – Cd_1 + AE group, and e – Cd_5 group are marked as $^{*}\ p < 0.05$, $^{\dagger}\ p < 0.01$, $^{\ddagger}\ p < 0.001$, and $^{\#}\ p = 0.05$. To evaluate the effect size for the differences between the experimental groups, eta squared (η^2) was calculated (for $\eta^2 \geq 0.14$ the effect size is large).
